# Enhanced antibacterial activity of antimicrobial peptide—antibiotic combinations against multidrug-resistant bacteria

**DOI:** 10.1093/femsmc/xtag003

**Published:** 2026-02-04

**Authors:** Muhammad Talha, Cesar Augusto Roque-Borda

**Affiliations:** Department of Pharmacy, COMSATS University Islamabad, Lahore Campus, Lahore 54000, Pakistan; Vicerrectorado de Investigación, Universidad Católica de Santa María, Arequipa 04000, Peru

**Keywords:** antimicrobial resistance, antimicrobial peptides, antibiotics, combination therapy, synergy, therapeutic approaches, resistance mechanisms

## Abstract

The rapid emergence of multidrug-resistant (MDR) bacteria has severely compromised the efficacy of conventional antibiotics and intensified the global antimicrobial resistance crisis. Antimicrobial peptides (AMPs) have attracted considerable interest as adjunctive agents due to their membrane-active mechanisms and immunomodulatory properties; however, their clinical use as monotherapy remains limited by instability, toxicity, and pharmacokinetic constraints. Combining AMPs with conventional antibiotics has emerged as a promising strategy to enhance antibacterial efficacy, restore antibiotic susceptibility, and modulate resistance development. This review critically examines the mechanistic basis of AMP–antibiotic synergy, integrating evidence from *in vitro* and *in vivo* studies. Particular emphasis is placed on determinants that govern synergistic outcomes, including membrane permeability, porin-dependent antibiotic uptake, resistance-associated adaptations, and host-related factors that cannot be captured *in vitro*. In addition, we discuss key translational barriers limiting clinical implementation, such as immune modulation, pharmacokinetic mismatch, peptide instability, and strain-dependent variability in synergistic responses. By linking molecular mechanisms to experimental and translational outcomes, this review provides a focused framework for rational design and optimization of AMP–antibiotic combination therapies against MDR bacterial infections.

## Teaser

This article explores how combining natural antimicrobial peptides with conventional antibiotics offers a powerful new strategy to overcome drug resistance and improve treatment of multidrug-resistant infections.

## Introduction

The alarming global rise of multidrug-resistant (MDR) bacterial infections is compromising the clinical utility of antibiotics and placing immense pressure on healthcare systems. In 2019 alone, antimicrobial resistance (AMR) directly accounted for ~1.27 million deaths, with projections estimating up to 10 million annual deaths by 2050 if no immediate interventions are implemented (Murray et al. 2022, Xu et al. 2024). Data from the WHO Global Antimicrobial Resistance and Use Surveillance System (GLASS) 2025 report indicate persistently high resistance levels among key bacterial pathogens, particularly in invasive infections reported across multiple regions, with resistance to third-generation cephalosporins observed in 44.8% of *Escherichia coli* bloodstream isolates, while methicillin resistance was reported in 27.1% of *Staphylococcus aureus* isolates globally (World Health Organization ). The growing inability of standard treatments—such as β-lactams and fluoroquinolones—to effectively manage common infections illustrates the limitations of the current antibiotic arsenal.

The efficacy of conventional antibiotics has progressively declined as a result of extensive misuse in clinical and agricultural settings, accelerated dissemination of resistance genes through horizontal gene transfer, and the scarcity of newly developed agents with novel mechanisms of action (Fang et al. 2024, Malik 2024). Most approved antibiotics are narrow spectrum, while resistance to broad-spectrum agents is accelerating (Khurana et al. 2024). In parallel, antibiotic-associated toxicities such as nephrotoxicity, ototoxicity, hepatotoxicity, and gastrointestinal disturbances add to the therapeutic burden (Lombardi et al. 2024). Moreover, conventional antibiotics often fail against biofilm-associated pathogens—such as *Pseudomonas aeruginosa, S. aureus*, and *Acinetobacter baumannii*—due to poor penetration, the presence of extracellular polymeric substances, quorum-sensing-mediated stress responses, differences in bacterial metabolic activity, drug tolerance, and rapid resistance development (Javanmard et al. 2024, Xiong et al. 2024, Upadhyay et al. 2025).

Other limiting factors include short drug half-lives requiring frequent dosing, increased risk of patient noncompliance, and high production costs. These constraints are further shaped by long-standing structural and economic disincentives affecting antibiotic development pipelines (World Health Organization 2025, Ge et al. 2025). Additionally, broad-spectrum antibiotics can disrupt the gut microbiota, promoting dysbiosis and increasing susceptibility to opportunistic pathogens such as *Clostridium difficile* (Queen et al. 2020, Taitz et al. 2025). Host-related and environmental factors can further inactivate antibiotics, compromising efficacy against priority MDR organisms, such as carbapenem-resistant *Enterobacterales* (CRE) and methicillin-resistant *S. aureus* (MRSA) (Li et al. 2024b, Mengistu et al. 2024, Zhou et al. 2024). These limitations have shifted the therapeutic paradigm from monotherapy toward rational combination strategies. In this context, antimicrobial peptides (AMPs) are particularly attractive partners for conventional antibiotics, as their rapid membrane-disruptive or immune-modulatory actions can sensitize bacteria to antibiotics that would otherwise be ineffective. Importantly, AMP–antibiotic synergy is not merely additive, but often mechanistically driven, enabling lower dosing, restoration of antibiotic susceptibility, and suppression of resistance emergence (Mahlapuu et al. 2016).

The molecular basis of AMR is diverse and deeply entrenched in microbial evolution. Antibiotic resistance genes, which predate the antibiotic era, are present in environmental reservoirs such as permafrost, soil, water, and hospital settings (Wang et al. 2021, Lee and Yoo 2022). These genes confer resistance to nearly all major antibiotic classes, including tetracyclines, aminoglycosides, beta-lactams, and fluoroquinolones (da Costa de Souza et al. 2022). Mechanistically, bacteria employ enzymatic degradation, efflux pumps, target site modifications, target replacement, and decreased membrane permeability to evade antibiotic action (Wang et al. 2021). This adaptive flexibility renders many first- and second-line therapies ineffective, thereby highlighting the limitations of conventional antibiotic monotherapy and reinforcing the need for alternative or complementary therapeutic strategies (Satchanska et al. 2024a, Mulukutla et al. 2024, Odunitan et al. 2024).

AMPs exhibit a wide range of mechanisms of action, charges, and structural features. While extensive studies are available on cationic and amphipathic AMPs interacting with negatively charged microbial membranes, some AMPs possess a net negative or neutral charge and exhibit antimicrobial activity against pathogens via ion sequestration, immune modulation, or enzyme inhibition (Satchanska et al. 2024a, Odunitan et al. 2024). Despite these advantages, the clinical translation of AMPs remains constrained by unfavorable pharmacokinetic properties, including short plasma half-lives and susceptibility to proteolytic degradation, reinforcing the need for combination strategies that reduce the required peptide exposure while preserving efficacy.

Combining AMPs with conventional antibiotics presents a viable strategy to overcome these limitations. Synergistic interactions have demonstrated enhanced bacterial killing, reduced dosing requirements, and delayed resistance emergence (Zharkova et al. 2019, Han et al. 2011). AMPs can increase membrane permeability, thereby facilitating antibiotic entry and improving intracellular targeting (Huang et al. 2024a). This combinatorial approach has shown efficacy against clinically relevant MDR pathogens, including CRE, MRSA, *P. aeruginosa, A. baumannii*, and *Klebsiella pneumoniae* (Bhattacharjya et al. 2024, Papazachariou et al. 2024, Raza et al. 2024, Tüzemen et al. 2024). Furthermore, such strategies align with the One Health framework adopted globally in 2015, which emphasizes integrated approaches to AMR through multisectoral collaboration, surveillance, and sustainable therapeutic development (World Health Organization 2023). Accordingly, this review critically examines the mechanistic basis, experimental evidence, and translational relevance of AMP–antibiotic synergy, with particular emphasis on how complementary modes of action can be exploited to combat MDR bacterial infections.

## AMPs and their relationship with antibiotics

AMPs are evolutionarily conserved molecules produced by a wide range of organisms, including bacteria, fungi, plants, invertebrates, and mammals, as part of their innate immune defense (Roque-Borda et al. 2026). In recent years, AMPs have gained increasing attention as therapeutic candidates due to their potent activity against MDR bacterial pathogens. Their clinical relevance stems from their ability to act through mechanisms that differ from those of conventional antibiotics, often involving membrane perturbation and interference with intracellular targets, which can complement antibiotic activity rather than replace it (Browne et al. 2020, Goki et al. 2024). Structurally, many AMPs are short, cationic, and amphipathic molecules that interact with negatively charged bacterial membranes, leading to pore formation, membrane depolarization, and loss of cellular integrity. Some AMPs can also penetrate bacterial cells and interfere with essential intracellular processes such as protein synthesis, nucleic acid function, or enzymatic activity. These multifaceted mechanisms contribute to their antibacterial activity and provide a mechanistic basis for their use in combination with conventional antibiotics, particularly against pathogens resistant to standard therapies (Dong et al. 2024, Wang et al. 2024).

Unlike traditional antibiotics that typically target specific bacterial enzymes or pathways, AMPs exert rapid and multitarget effects that can enhance bacterial susceptibility to coadministered antibiotics. Rather than preventing resistance outright, these properties may reduce the selective pressure associated with antibiotic monotherapy and help delay resistance emergence when used in rational combination strategies (Huang et al. 2024b, Kanaujia et al. 2024). In this context, AMPs represent a mechanistically distinct and clinically valuable component of the antimicrobial arsenal, especially against high-priority pathogens identified by the World Health Organization. Although their clinical translation remains challenged by issues related to stability, bioavailability, and potential cytotoxicity, ongoing structural optimization and formulation approaches are increasingly positioning AMPs as effective synergistic partners to conventional antibiotics rather than standalone replacements.

### Mechanism of action of antibiotics and bacterial resistance mechanism against antibiotics

**Figure 1:**
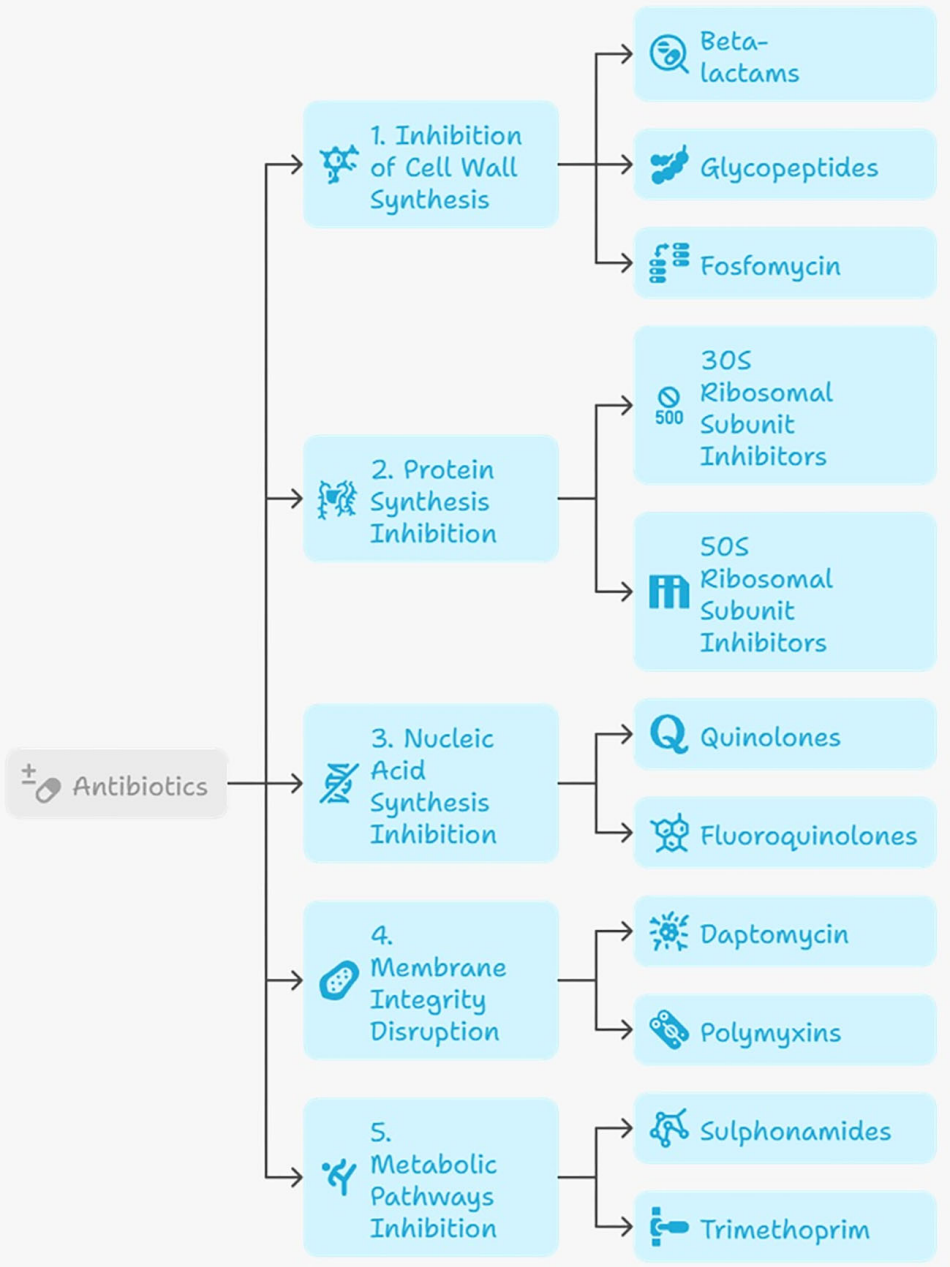
Classification of antibiotics based on mechanism of action. The figure summarizes major antibiotic classes and their corresponding mechanisms: (1) inhibition of cell wall synthesis (e.g. β-lactams, glycopeptides, and fosfomycin); (2) protein synthesis inhibition via binding ribosomal subunits (50S and 30S inhibitors), including macrolides, aminoglycosides, oxazolidinones, and tetracyclines; (3) nucleic acid synthesis inhibition (quinolones and fluoroquinolones); (4) membrane disruption (including daptomycin and polymyxins); and (5) inhibition of metabolic pathways (sulfonamides and trimethoprim) (Etebu and Arikekpar 2016, Roque-Borda et al. 2021).

Antibiotics can be classified according to their chemical structure, antimicrobial spectrum, or mechanism of action, the latter being the most relevant from a clinical and microbiological perspective (Fig. 1). Functionally, antibiotics are grouped according to the bacterial processes they inhibit, including cell wall synthesis, protein synthesis, nucleic acid synthesis, folate-dependent metabolic pathways, and membrane integrity. This classification facilitates understanding of therapeutic applications and provides a framework for anticipating resistance mechanisms.

Cell wall synthesis inhibitors include β-lactams—such as penicillins, cephalosporins, monobactams, and carbapenems—and glycopeptides such as vancomycin. These antibiotics interfere with peptidoglycan biosynthesis, a process essential for bacterial viability, particularly in Gram-positive organisms. β-lactams act by inhibiting penicillin-binding proteins (PBPs), enzymes involved in the transpeptidation step of cell wall cross-linking, thereby compromising structural integrity and causing lysis (Egorov et al. 2020, Lima et al. 2020). Glycopeptides and fosfomycin also inhibit cell wall synthesis but act at different stages of peptidoglycan production. Glycopeptides bind the d-Ala–d-Ala terminus of peptidoglycan precursors, blocking incorporation into the cell wall, whereas fosfomycin targets MurA and inhibits an early cytoplasmic step in cell wall biosynthesis (Lade and Kim 2021).

Protein synthesis inhibitors exploit structural differences between bacterial and eukaryotic ribosomes by selectively binding bacterial ribosomal subunits. Aminoglycosides (e.g. gentamicin and streptomycin) and tetracyclines (e.g. doxycycline) bind the 30S subunit, interfering with tRNA binding and decoding, while macrolides (e.g. azithromycin), lincosamides, and chloramphenicol inhibit protein synthesis via interactions with the 50S subunit (Lade and Kim 2021). Quinolones and fluoroquinolones, such as ciprofloxacin and moxifloxacin, inhibit nucleic acid synthesis by targeting bacterial type II topoisomerases (DNA gyrase and topoisomerase IV). This leads to replication fork stalling and double-strand DNA breaks; some studies further suggest that DNA damage and subsequent generation of reactive oxygen species may contribute to bacterial killing, particularly under aerobic conditions (El-Fateh et al. 2024).

Metabolic pathway inhibitors, particularly sulfonamides and trimethoprim, target successive steps in folate biosynthesis. Sulfonamides inhibit dihydropteroate synthase, while trimethoprim inhibits dihydrofolate reductase, collectively blocking tetrahydrofolate production and impairing nucleotide biosynthesis (Whalen 2018). Membrane integrity disruptors, such as polymyxins (polymyxin B and colistin), exert rapid bactericidal activity by binding to lipopolysaccharide (LPS) in the outer membrane of Gram-negative bacteria. This interaction disrupts membrane architecture and increases permeability, ultimately leading to cell death (Padhy et al. 2024).

Understanding these mechanistic classes informs rational antibiotic selection and highlights vulnerabilities that bacteria exploit to develop resistance. The following section outlines the molecular basis of these resistance mechanisms and their relationship to the antibiotic classes described above. The growing inefficacy of conventional antibiotics is largely driven by bacterial resistance, which can be intrinsic or acquired. Intrinsic resistance reflects inherent structural or functional features that limit antibiotic activity, such as low membrane permeability or constitutive efflux pump expression. Acquired resistance arises through genetic mutations or horizontal gene transfer, enabling bacteria to inactivate drugs, modify targets, reduce intracellular drug concentrations, or bypass affected pathways (Darby et al. 2023). The most prevalent and mechanistically well-characterized resistance strategies relevant to antibiotic activity are summarized in Table 1.

**Table 1:** Core molecular mechanisms driving bacterial antibiotic resistance.

Resistance mechanism	Molecular basis	Representative pathogens	Affected antibiotic classes	Key references
Reduced intracellular drug accumulation	Porin loss (e.g. OprD); efflux pump overexpression (AcrB, MdeA, and AdeABC)	*P. aeruginosa, E. coli, S. aureus, A. baumannii*	Carbapenems, aminoglycosides, macrolides, fluoroquinolones, and tetracyclines	Floyd et al. (2010), Schuster et al. (2021), Dodan et al. (2023), Sinha et al. (2024), Vattanaviboon et al. (2025)
Target modification by mutation	Mutations in gyrA/parC; altered PBPs (mecA/mecB)	*E. coli, S. aureus*	Fluoroquinolones and β-lactams	Turner et al. (2019), Kamoshida et al. (2020)
Target protection/ribosomal methylation	23S rRNA methylation (erm genes)	*S. aureus, Streptococcus* spp.	Macrolides, lincosamides, and streptogramins	Babosan et al. (2022), An et al. (2023), Muhs et al. (2025)
Enzymatic drug inactivation	ESBLs; carbapenemases (KPC, NDM, VIM, OXA, and IMP)	Enterobacterales, *P. aeruginosa*	β-lactams and carbapenems	Bandy and Tantry (2021), Beshah et al. (2023), Huang and Li (2023)

### AMPs

AMPs are naturally occurring molecules that play a central role in innate immunity across a wide range of organisms and display activity against bacteria, viruses, fungi, and parasites (Oliveira Júnior et al. 2025). Their relevance as antimicrobial agents stems from their ability to act against antibiotic-resistant pathogens and to engage mechanisms that differ fundamentally from those of conventional antibiotics. Structurally, AMPs are generally short peptides (<10–100 amino acids) that often possess cationic and amphipathic features, enabling preferential interactions with negatively charged microbial membranes (Roque-Borda et al. 2025c). These interactions can lead to membrane destabilization through supramolecular arrangements such as toroidal, barrel-stave, carpet, or detergent-like mechanisms, ultimately compromising membrane integrity (Pfalzgraff et al. 2018). In addition to membrane-associated effects, many AMPs exert intracellular activities, including interference with nucleic acid synthesis, cell wall assembly, cell division, and essential enzymatic pathways.

Compared with conventional antibiotics, AMPs are characterized by rapid antimicrobial activity, functional versatility, and immunomodulatory properties. Importantly, their mechanisms of action intersect with several resistance-associated vulnerabilities described for antibiotics, providing a mechanistic rationale for their use as synergistic partners in combination therapies. The major classification schemes of AMPs, based on source, functional activity, amino acid composition, and structural features, are summarized in Table 2.

**Table 2.** Principal classification frameworks for antimicrobial peptides based on source, function, composition, and structure.

Classification criterion	Category / Class	Defining features	Representative peptides	Reference
Source-based	Mammals	Mainly cathelicidins and defensins; immunomodulatory and antimicrobial roles	LL-37, β-defensin-2	(Lakshmanan, Nair and Swathi Prabhu 2023)
	Amphibians	High structural diversity; potent membrane activity	Magainins, aurein	(Li et al. 2022)
	Insects / Arthropods	Synthesized in hemolymph and fat bodies; broad-spectrum activity	Cecropins, jelleins	(Shelomi et al. 2020; Zahedifard et al. 2020)
	Plants	Often cysteine-rich; stable in harsh environments	Thionins, defensins, snakins	(Li et al. 2021)
	Microbial (bacteria/fungi)	Often ribosomally synthesized; some clinically approved	Gramicidin, nisin	(Jhong et al. 2022)
	Marine organisms	Emerging and underexplored AMP reservoir	As-CATH4, Myticusin-β	(Semreen et al. 2018; Oh et al. 2020)
Functional	Antibacterial	Membrane disruption or intracellular targeting of bacteria	P5, P9	(Li et al. 2019)
	Antifungal	Target fungal membranes, cell wall, or intracellular processes	AurH1, plant AMPs	(Roque‐Borda et al. 2025)
	Antiviral	Interfere with viral entry or replication	Defensins, cathelicidins	(Yu et al. 2021; Guo et al. 2023)
	Antiparasitic	Activity against protozoa and helminths	Cecropins	(Lou et al. 2024)
	Disease-associated / immunomodulatory	Modulate inflammation and host immune response	LL-37, smaragin	(Feng et al. 2025)
Amino acid composition	Proline-rich peptides (PrAMPs)	Enter cytoplasm; inhibit ribosomal function	Bac7, Tur1A	(Mardirossian et al. 2018, 2019)
	Arginine- and tryptophan-rich	Strong membrane interaction via ion-pair π interactions	Melittin-Thanatin	(Liu et al. 2023)
	Glycine-rich peptides	Often non-lytic; diverse biological roles	Attacins, diptericins	(Wang et al. 2024)
	Histidine-rich peptides	pH-responsive; antibiofilm and immunomodulatory	D-histidine, HV2	(Dong et al. 2019; Lointier et al. 2020)
Structural features	α-helical	Amphipathic; strong membrane activity	LL-37, magainin	(Pfalzgraff et al. 2018)
	β-sheet / cysteine-rich	Disulfide-stabilized; protease-resistant	Defensins	(González‐Castro, Gómez‐Lim and Plisson 2021)
	Extended / non-helical	Flexible conformations; intracellular targets	Indolicidin	(Mitra et al. 2024)
	Cyclic peptides	High stability; reduced proteolysis	lantibiotics	(Joo et al. 2016).

### Mechanisms of AMP activity and bacterial resistance

AMPs exert their activity through a wide array of mechanisms that target microbial viability and virulence. While multiple pathways have been described—including interference with nucleic acid synthesis, enzyme inhibition, and disruption of quorum sensing—most well-characterized amps act via three principal routes: membrane disruption, inhibition of cell wall synthesis, and modulation of host immune responses (Magana et al. 2020). These core mechanisms are not mutually exclusive and often operate synergistically within the same peptide molecule. Below, we detail these modes of action, which not only define the functional landscape of amps but also guide their clinical translation and rational design. These mechanisms are central to understanding how AMPs enhance antibiotic activity, either by increasing bacterial permeability, exposing intracellular targets, or modulating host responses that influence treatment outcomes. A mechanistic understanding of AMP activity is therefore essential for predicting and rationalizing AMP–antibiotic synergy. Following are classification and mechanism of action of AMPs based on source Table 3.

**Table 3.** Representative antimicrobial peptides categorized by biological source and experimentally supported mechanisms of action.

Types of AMPs	Example			
Microbial AMPs	Name	Source	Mechanism of action	Reference
	Nisin	*Lactococcus lactis*	Membrane disruption: Target bacterial (14C)-Lipid II form complex led to formation of pore.	(Radaic, de Jesus and Kapila 2020; Maurya et al. 2023)
	Polymyxin	*Paenibacillus polymyxa*	Membrane disruption: Destabilize membrane structure by binding lipopolysaccharides, displacing essential divalent cation, increasing permeability.	(Kaye, Pogue and Kaye 2015; Qian, Mobashery and Fisher 2023)
	Gramicidin	*B. brevis*	Disrupts bacterial cell membrane by formation of ion channels leading to the loss of ion and act as ionophore.	(Stein and Litman 2015)
	Mersacidin	*Bacillus subtilis*	Cell Wall synthesis Inhibitor: Interacts lipid II, stops transglycosylation step thus inhibits cell wall synthesis.	(Chattopadhyay, Das and Banerjee 2023)
	Microcin L	*E. coli*	Is non-pore forming peptide, require Cir receptor on microbial membrane to enter, disrupts TonB protein ultimately disrupts proton- motive forces and thus could lead to *E. coli* membrane depolarization.	(Morin et al. 2011; Jha 2024)
	Duramycin	*Streptomyces duramitus*	Membrane disruption: bind phosphatidylethanolamine (phospholipid) disrupts integrity of cell membrane, also target macromolecules synthesis pathways like nucleic acid duplication, protein synthesis and cell wall synthesis.	(Yuan et al. 2025)
	PLNC αβ	*L. plantarum*	Disrupt membrane of *P. gingivalis*	(Khalaf et al. 2016)
	Plectasin	*Pseudoplectania nigrella*	Inhibit bacterial cell wall by trapping lipid II in membrane. Immunomodulatory effect by producing interferon γ and serum IgY level.	(Schneider and Sahl 2010)
Plant AMPs	Snaking-Z	*Zizipus jujuba* (Chinese date)	Disrupts cell membrane and inhibits acetylcholinesterase and butyrylcholinesterase enzymes	(Kumar et al. 2025)
	α1-purothionin	*Triticum aestivum* (common or bread wheat)	Isolated from endospore of wheat, studies suggested that they can form pores when incorporated into the liposomes. Forming ion channels leading to the induction in membrane depolarization.	(Llanos et al. 2006)
	PvD1	*Phaseolus vulgaris* (Common bean)	Exhibit its antimicrobial action by increasing the production of nitric oxide, reactive oxygen species and disrupt microbial membrane of yeast and fungi.	(do Nascimento et al. 2015; de Oliveira Mello et al. 2019)
	Kalata B1	*Oldenlandia affinis* (Blue diamond flower)	Exhibits their activity by insertion of oligomers in lipid bilayer forming pore thus increasing the membrane permeability.	(Huang et al. 2009; Nawae, Hannongbua and Ruengjitchatchawalya 2014)
	Hevein	*Hevea barsiliensis* (Sharinga tree or rubber tree)	Obtained from rubber latex, it exhibits its activity by binding to the N-acetyl-D-glucosamine of fungal cell wall (chitin) causing inhibition of cell wall synthesis.	(de Souza Cândido et al. 2014)
	WjAMP1	*Wasabia japonica* (Japanese horseradish)	Inhibits fugal growth by binding to chitin (cell wall) of *Botrytis cinerea*.	(Yamuna et al. 2019)
Animals and Insects AMPs	IB-367 (Iseganan)	Porcine (pigs) leukocytes	Disrupt the cell membrane integrity if bacteria and fungi by targeting lipid cell membrane.	(Toney 2002; Rios et al. 2016)
	BMAP-28	Bovine Myeloid (*Bos taurus*)	Electron microscopic studies suggested that AMP target bacterial cell membrane leading to permeation. Studies also suggested its activity by inhibiting of biofilm of *C. albicans*.	(Agadi, Vasudevan and Kumar 2018; Talapko et al. 2022)
	Protegrin-1	Porcine (pigs) leukocytes	Exhibits is activity by disruption and increasing the permeability of bacterial membrane. Also, Protegrin 1 show immune modulatory potential neutralizing the activation of bacteria-induced RAW macrophages and lipopolysaccharides by binding and prevents the cell surface lipopolysaccharides attachments. And inhibit the survival of amastigotes in leishmania infection.	(Javed et al. 2024)
	Magainin-2	Skin secretion *Xenopus laevis*	It forms pores in lipid bilayer via toroidal mechanism, increasing the cell permeability ultimately cell death.	(Di Somma et al. 2022)
	Buforin II	*Bufo bufo gargarizans-*Stomach tissue of Asian toad	Exhibit its activity without permeabilizing the membrane instead it enters cell form condensate with microbial DNA, interfering cellular structures and processes, leading to cell death without membrane permeation.	(Tolos (Vasii) et al. 2024)
	Temporin A	*Rana temporaira* (Skin secretion of European red frog)	Membrane disruption- via amphipathic α-helical mechanism.	(D’Andrea and Romanelli 2023)
	Citropin 1.1	*Litoria citropa* (Skin secretion of Australian tree frog)	Membrane disruption via carpet model –conformational changes occur in α-helical structure that integrate with lipid membrane, cause membrane permeation.	(de Almeida et al. 2019; Li et al. 2022)
	Aurein 1.2	*Litoria aurea* (Granular skin of Australian tree frog)	Disrupts bacterial cell membrane via carpet model. Another study suggests that aurein 1.2 inhibit the biofilm formation up to 80% of *Candidia parapsilosis*.	(Raheem et al. 2020; Silva et al. 2025)

#### Membrane disruption mechanism

Among the diverse mechanisms through which AMPs exert their activity, membrane disruption remains the most immediate and consistently observed. In bacterial systems, the process is typically initiated through electrostatic interactions between cationic peptides and the anionic components of the microbial membrane, particularly phospholipid headgroups (Benfield and Henriques 2020). The interaction between AMPs and lipid bilayers is influenced by a complex interplay of physicochemical properties. Key factors include the peptide’s net charge, amphipathicity, secondary structure, concentration, and the nature of the target membrane. Notably, the outcome of these interactions is not solely determined by peptide structure, but by the contextual balance between peptide and lipid stoichiometry, membrane composition, ionic strength, and environmental pH (Zhang et al. 2021).

Several models have been proposed to describe the physical outcome of AMP–membrane interactions, classically divided into pore-forming and nonpore-forming categories. In the barrel-stave model, exemplified by peptides such as alamethicin (Goki et al. 2024). A mechanistically distinct variant, the toroidal-pore model, involves peptides like magainin and LL-37 (Volovik and Batishchev 2024). Both models result in uncontrolled ion flux, leakage of cytoplasmic content, and cell death, but differ in the structural reorganization of the membrane components.

Not all peptides, however, follow a pore-based mechanism. In many cases, AMPs accumulate on the membrane surface in a parallel orientation, acting cooperatively to induce destabilization without forming discrete pores. This so-called carpet model, described for dermcidin and indolicidin (Zhang et al. 2021). A closely related, though more physiochemically grounded, mechanism is the detergent-like model, in which peptides such as tritrpticin, gramicidin S, or synthetic peptoids behave as amphipathic surfactants, extracting lipids from the bilayer and promoting micelle formation (Goki et al. 2024). Although structurally distinct from the pore-forming models, both nonpore mechanisms lead to the same endpoint: catastrophic loss of membrane integrity.

**Figure 2:**
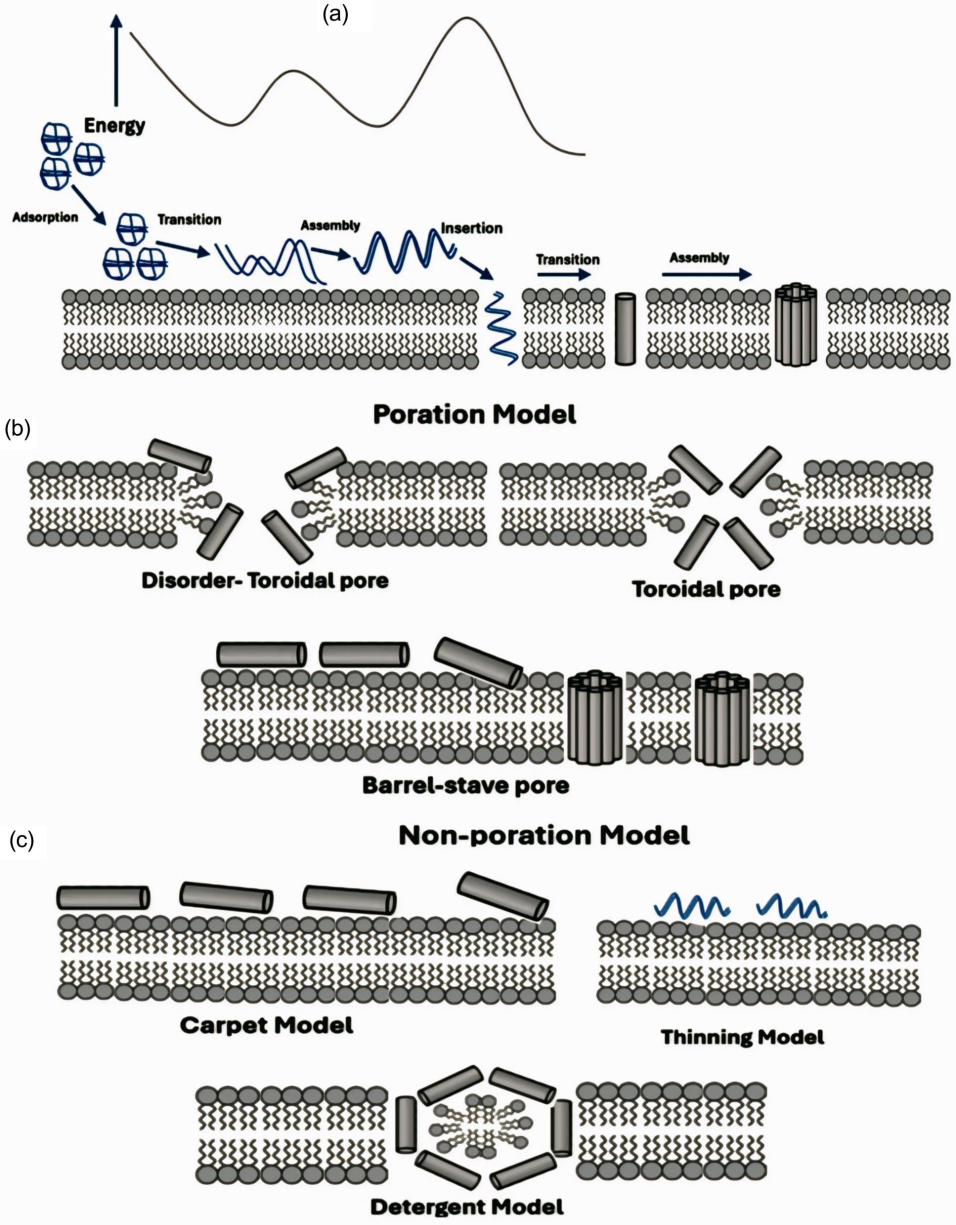
Mechanisms of AMP-induced membrane disruption. (a) Sequential steps of AMP interaction with membranes, including adsorption, conformational transition, insertion, and assembly. (b) Pore-forming models: barrel-stave, toroidal, and disordered toroidal pores. (c) Nonpore models: carpet, thinning, and detergent-like mechanisms leading to membrane disintegration without defined pore formation.

In addition to defining distinct structural models, AMP–membrane interactions follow a dynamic and energetically regulated sequence that underlies membrane disruption. As illustrated in Fig. 2(a), this process typically begins with electrostatic adsorption to the membrane surface, followed by peptide conformational transitions, cooperative assembly, and eventual insertion into the lipid bilayer. The energetic landscape of these steps determines whether peptides form stable transmembrane structures or induce membrane destabilization through nonporating mechanisms. Within pore-forming pathways (Fig. 2b), AMPs may generate barrel-stave pores or toroidal pores, including disordered toroidal intermediates characterized by transient lipid–peptide rearrangements. In contrast, nonporating mechanisms (Fig. 2c), such as carpet, thinning, and detergent-like models, involve surface accumulation and lipid extraction without the formation of discrete channels, ultimately leading to loss of membrane integrity.

#### Cell wall synthesis inhibition mechanism

While the disruption of bacterial membranes remains the most immediate and broadly observed action of AMPs, a subset of these molecules also interferes directly with cell wall biosynthesis. This mechanism targets peptidoglycan, a core structural component that maintains bacterial shape and withstands osmotic pressure. Several AMPs bind to lipid II—a conserved precursor in peptidoglycan synthesis—thereby blocking its incorporation into the growing cell wall. Others interfere with enzymes involved in remodeling or cross-linking, undermining the structural integrity of the cell wall and sensitizing bacteria to lysis (Huang and Li 2023). This mode of action is particularly effective against Gram-positive bacteria, where lipid II is readily accessible at the outer leaflet of the cytoplasmic membrane. In Gram-negative species, access is limited by the outer membrane; however, certain peptides can overcome this barrier through LPS destabilization or translocation via porin proteins, enabling access to periplasmic targets. Disruption of these processes ultimately impairs normal cell wall synthesis and bacterial viability.

#### Immune modulation by AMPs

In addition to their direct bactericidal activity, many AMPs—also referred to as host defense peptides (HDPs)—play key roles in modulating the host immune response. This dual function represents a strategic evolutionary advantage, as it allows AMPs not only to eliminate pathogens but also to shape the inflammatory environment and influence immune homeostasis (Guryanova and Ovchinnikova 2022). AMPs modulate innate and adaptive immunity through multiple mechanisms. They can regulate the expression of proinflammatory and antiinflammatory cytokines, enhance chemotaxis, and influence the differentiation and activation of immune cells including macrophages, dendritic cells, neutrophils, and T lymphocytes (Drayton et al. 2021). Some peptides also suppress excessive inflammatory responses by downregulating the release of cytokines such as TNF-α, IL-6, or IL-1β, and by neutralizing endotoxins like LPS, thereby reducing the risk of sepsis or tissue damage. This immunoregulatory potential is particularly relevant in infectious settings, where dysregulated host responses significantly contribute to disease severity.

One illustrative example is porcine β-defensin 2, a mammalian AMP homologous to human defensins, which has been shown to inhibit the TLR/NF-κB signaling pathway. This inhibition reduces the expression of inflammatory mediators and improves survival outcomes in infection models involving Pseudorabies virus, highlighting a broader immune-protective role that extends beyond direct antimicrobial activity (Huang et al. 2020, Zhang et al. 2021).

### Bacterial resistance to AMPs

**Figure 3:**
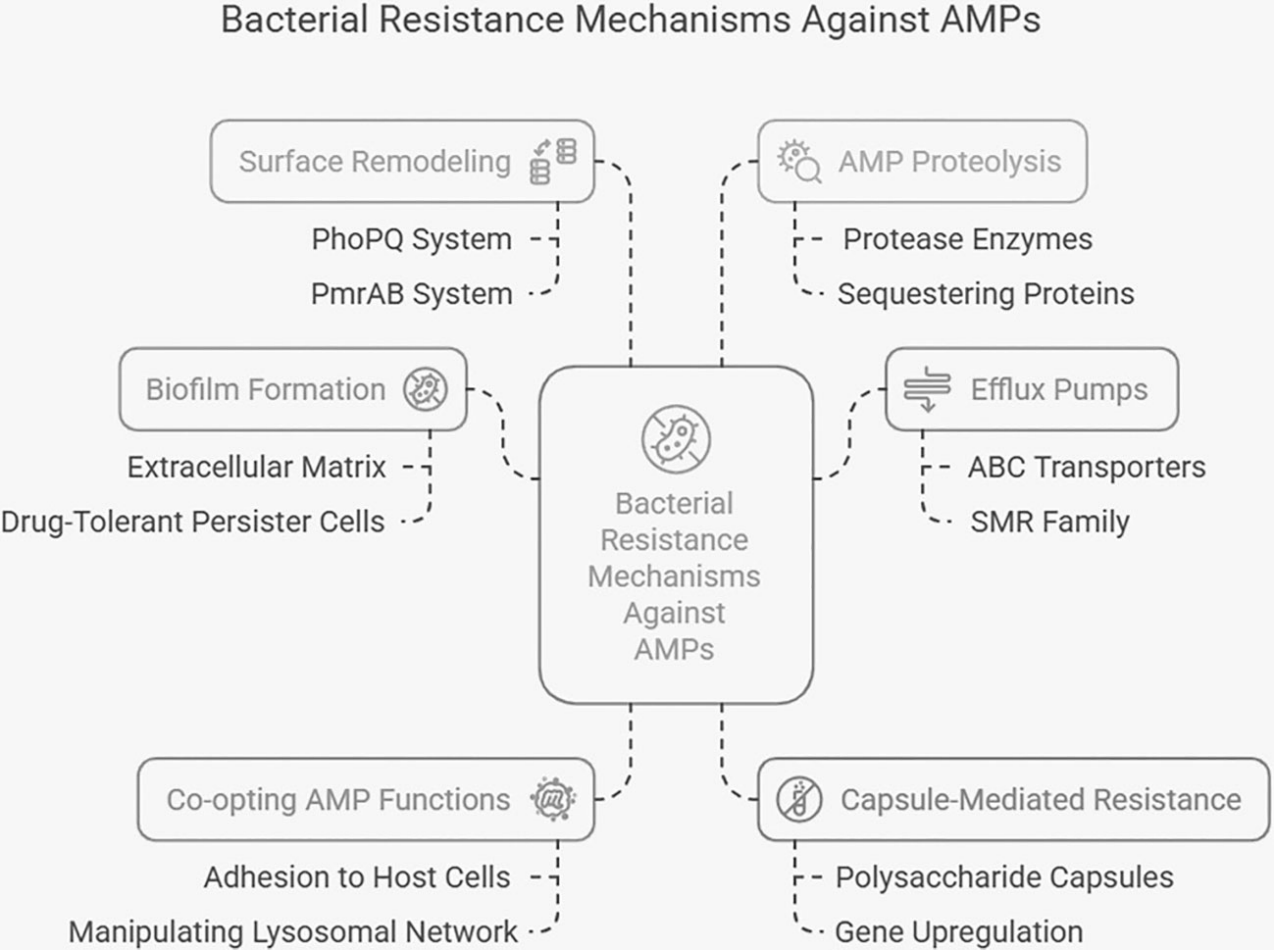
Overview of major bacterial strategies that reduce AMP efficacy. These include surface remodeling mediated by TCSs (e.g. PhoPQ and PmrAB), biofilm formation with extracellular matrix production and persister cell development, AMP proteolysis and extracellular sequestration, efflux pump activity, and capsule-mediated resistance. In addition, certain pathogens can functionally coopt host-derived AMPs to enhance adhesion or intracellular survival, indirectly limiting their antimicrobial effectiveness.

AMPs are key effectors of the innate immune system, exerting their activity by disrupting membranes or targeting intracellular components and metabolic pathways (Shriwastav et al. 2025). However, highly adaptable bacterial pathogens have evolved multiple strategies to counteract AMP activity. Unlike conventional antibiotics, resistance to AMPs often arises from structural and regulatory adaptations, including capsule synthesis, surface remodeling to reduce peptide binding, production of AMP-sequestering proteins, biofilm formation, efflux pump activation, membrane transport modifications, and proteolytic degradation. In some cases, bacteria may even coopt AMP functions to their own advantage. These mechanisms, summarized in Fig. 3, underscore the complexity of AMP resistance and highlight the challenges associated with their therapeutic translation.

#### Biofilm formation

Biofilms are structured bacterial communities embedded in a self-produced extracellular polymeric matrix composed of DNA, lipids, polysaccharides, and proteins, which provides both genetic and physicochemical protection against AMPs (Gan et al. 2021). Biofilm formation is a central mechanism underlying bacterial resistance to both AMPs and conventional antibiotics, as the dense extracellular matrix restricts peptide penetration (World Health Organisation 2020). Moreover, biofilms create a protective niche for drug-tolerant persister cells, prolonging resistance and further compromising AMP efficacy. Biofilm-associated bacteria also counteract AMPs by upregulating proteases, efflux pumps, and quorum-sensing pathways (Schulze et al. 2021). The bacterial response to AMPs within biofilms is highly dependent on peptide composition, structure, and concentration (Duperthuy 2020). For example, increasing concentrations of AMP-17 have been shown to disrupt preformed biofilms or prevent *Candida albicans* biofilm formation (Sun et al. 2022, Xuan et al. 2023). Importantly, recent studies emphasize that through rational design and structural optimization, AMPs can act as effective biofilm inhibitors—sometimes functioning not primarily as antimicrobials but rather as agents targeting biofilm formation or associated virulence mechanisms. A well-documented review further highlights these findings and supports the development of AMPs as promising tools against recalcitrant pathogens (Roque‐Borda et al. 2024).

#### Efflux pumps/membrane transport system mechanism

Efflux pumps are among the most conserved bacterial defense mechanisms against antimicrobials, functioning as membrane transport systems that actively expel toxic compounds. The major families implicated in AMP resistance include the ATP-binding cassette (ABC) transporters, the small multidrug resistance family, the RND family, the multidrug and toxin extrusion family, and the major facilitator superfamily (Jorgensen et al. 2023, Revol-Tissot et al. 2024, Sagar and Priti 2024). These systems are frequently upregulated in response to antibiotics or host immune factors and can be further enhanced by horizontal gene transfer or point mutations (Fang et al. 2024). By exporting antibiotics and AMPs, they reduce intracellular accumulation, while also contributing to virulence by promoting biofilm formation and regulating quorum sensing (Kaur et al. 2021). Experimental data illustrate these effects: in *E. coli*, sublethal exposure to brevinin-2CE altered the expression of AcrZ and SugE efflux proteins, leading to peptide expulsion and loss of activity. Notably, when brevinin-2CE was combined with chlorpromazine—an efflux pump inhibitor—antimicrobial activity increased up to five-fold, reducing bacterial resistance (Bing et al. 2024). Likewise, sublethal concentrations of pexiganan and melittin induced adaptive resistance in *E. coli* (Rodríguez-Rojas et al. 2021). These findings emphasize that efflux systems remain a major barrier to AMP efficacy and that combining AMPs with efflux inhibitors or designing efflux-resistant analogues could be critical for therapeutic translation.

#### Capsule-mediated resistance

Polysaccharide capsules are another widespread strategy conferring protection against AMPs. The negatively charged components of the capsule bind cationic peptides via electrostatic interactions, preventing them from reaching the bacterial membrane. In *Neisseria meningitidis*, the capsule not only acts as a barrier to LL-37 but is also transcriptionally upregulated at sublethal peptide concentrations through activation of *siaC* and *siaD*, strengthening resistance (Jones et al. 2009). Comparative studies in *Bacillus anthracis* further confirmed this effect: encapsulated wild-type strains displayed significantly greater resistance to defensins and other cationic AMPs compared with capsule-deficient *capA* mutants (O’Brien et al. 2022). Thus, capsule production simultaneously provides a physical shield and an inducible resistance mechanism that reduces AMP accessibility and antimicrobial potency.

#### Surface remodeling

Surface remodeling allows bacteria to reduce AMP binding and penetration by modifying membrane or cell wall composition. Gram-positive pathogens such as *S. aureus* and Gram-negative *E. coli* have been observed to thicken their cell walls through d-alanylation of teichoic acids, which decreases net negative charge and increases structural density (Maria-Neto et al. 2012). In Gram-negative species, two-component regulatory systems (TCSs) such as *PhoP–PhoQ* (*PhoPQ*) and *PmrA–PmrB* (*PmrAB*) are central regulators of outer membrane remodelling. Studies in *Salmonella enterica* demonstrated that disruption of PhoPQ increased susceptibility to AMPs, yielding a more permeable and negatively charged outer membrane, whereas disruption of *PmrAB* produced a more rigid, less permeable phenotype without altering AMP susceptibility (May and Groisman 2013, Pranantyo et al. 2022). These results highlight the dynamic and context-dependent nature of bacterial surface remodeling, where shifts in regulatory balance between TCSs fine-tune membrane flexibility and resistance capacity, ultimately enabling survival in hostile environments.

#### AMP proteolysis and extracellular sequestering proteins

Proteolytic degradation and extracellular sequestration are highly effective bacterial mechanisms to neutralize AMPs. For example, group A *Streptococcus* activates plasminogen through streptokinase (Ska), resulting in LL-37 degradation (Hollands et al. 2012). *Staphylococcus aureus* secretes multiple proteases, including endopeptidases such as V8 protease and metalloproteases such as aureolysin and *SepA*, which cleave LL-37 and other cathelicidins. Additionally, *S. aureus* secretes staphylokinase, encoded by the sak gene, which binds and sequesters human α-defensins (HNP-1 and HNP-2) (Gan et al. 2021, Garvey 2023). Other streptococci also deploy extracellular AMP inhibitors: *S. pyogenes* expresses streptococcal inhibitory components and M1 protein to neutralize defensins and LL-37, while *S. agalactiae* uses the pilus subunit PilB to bind cathelicidins and resist CRAMP and LL-37. Importantly, AMP susceptibility to proteolysis is structure dependent. Linear peptides such as LL-37 are highly vulnerable, whereas cyclic peptides stabilized by disulfide bonds, such as lantibiotics, show greater resistance (Joo et al. 2016). These findings indicate that protease secretion and peptide sequestration not only protect bacteria against innate immunity but also present significant obstacles to AMP-based therapeutics.

## Evidence of synergistic interactions from *in vitro* and *in vivo* studies

### *In vitro* synergistic interaction of antibiotic-AMPs

The rise of MDR pathogens poses a major threat to modern medicine, limiting the efficacy of conventional antibiotics, increasing the burden on healthcare systems, and contributing to the global prevalence of infectious diseases (Zhu et al. 2022, Taheri-Araghi 2024). AMPs have emerged as promising therapeutic candidates owing to their unique mechanisms of action, broad-spectrum activity, and comparatively lower risk of resistance development (Satchanska et al. 2024b). Nevertheless, their clinical use as monotherapy is restricted by susceptibility to enzymatic degradation, high production costs, and potential cytotoxicity (Mulukutla et al. 2024). To address these limitations, numerous *in vitro* studies have explored combinations of AMPs with conventional antibiotics. These combinatorial approaches not only improve efficacy against resistant strains but also delay resistance emergence, reduce dosing frequency, enhance pathogen killing, and minimize antibiotic-associated toxicities. However, while these *in vitro* studies provide valuable mechanistic and proof-of-concept insights, their translational relevance must be validated in appropriate *in vivo* models. A summary of representative *in vitro* studies evaluating AMP–antibiotic synergy is presented in Table 4.

**Table 4.** *In-vitro* studies reporting synergistic interactions between antimicrobial peptides and conventional antibiotics.

Antibiotics	AMPs	Target pathogens	Ref.
Novobiocin, Tigecycline	WLBU2	*Yersinia pestis*	(Cote et al. 2020)
Imipenem, Ciprofloxacin and Colistin	Melittin	*A. baumannii*	(Bardbari et al. 2018)
Tobramycin, Colistin, Doripenem, Ciprofloxacin, Ceftazidime	Nisin + P10	*A. baumannii*, Colistin-resistant *P. aeruginosa*	(Jahangiri et al. 2021)
Doripenem	Melittin	MDR *P. aeruginosa, A. baumannii*	(Akbari et al. 2019)
Aztreonam, Meropenem, rifampicin, tobramycin	SET-M33	*A. baumannii, P. aeruginosa, K. pneumoniae*	(Pollini et al. 2017)
Imipenem	AMP38	Imipenem-resistant *P. aeruginosa*	(Rudilla et al. 2016)
Ampicillin, Oxacillin	T3, T4	Methicillin-resistant *S. aureus*	(Rishi et al. 2018)
Polymyxin	Nisin	XDR pathogens, multidrug-resistant *A. baumannii*	(Thomas et al. 2019)
Carbapenem, Meropenem	P5	Carbapenem-resistant *P. aeruginosa*	(Martinez et al. 2019)
Gentamicin, Polymyxin	Pegylated LyeTx I-b	Carbapenem-resistant *A. baumannii, S. aureus*.	(César Moreira Brito et al. 2021)
Vancomycin, Rifampicin, Erythromycin.	PapMA-3	Carbapenem-resistant *A. baumannii*	(Choi et al. 2021)
Clindamycin	Melittin	Methicillin-resistant *S. aureus*	(Mahmoudi, Alikhani and Imani Fooladi 2020)
Rifampicin, Novobiocin, Vancomycin	WW307	MRSA T114, *E. coli*,	(Shi et al. 2021)
Azithromycin, Vancomycin, Chloramphenicol	G3KL	*P. aeruginosa, K. pneumonia*	(Gan et al. 2020)
Thiamphenicol, Florfenicol	LL-37, (p-BthTX-I )_2_	*Citrobacter freundii*	(Assane et al. 2021)
Chloramphenicol, Meropenem, rifampicin, ceftazidime	K11	MDR/XDR *K. pneumoniae*	(Chatupheeraphat et al. 2023)
Colistin, Carbapenem	ChIP	*Enterococcus faecium, S. aureus, K. pneumonia, A. baumannii, P. aeruginosa, E. coli*.	(Raza et al. 2024)
Meropenem, Colistin	LyeTx I	Carbapenem-resistant *A. baumannii*.	(Lima et al. 2021)
Colistin	MSI-78, OTD-244	*K. pneumoniae, A. baumannii, P. aeruginosa, E. coli*	(Witherell et al. 2020)
Halicin	SAAP-148	*S. aureus, E. coli*.	(van Gent et al. 2022)
Teicoplanin	LL-37, TC84, SAAP-276, SAAP-148	*S. aureus*	(Koppen et al. 2019)
Chlorhexidine digluconate (CHXD)	Platelet microbial proteins (PMP)	*S. aureus*	(Ivanov, Gritsenko and Kuzmin 2015)
Sulbactam	SAAP-148_M15	XDR *A. baumannii*	(Naha and Ramaiah 2024)
Ciprofloxacin	Melimine/MeI4	*S. aureus* ATCC 25923	(Yasir, Dutta and Willcox 2021)
Ceftazidime, Cefuroxime, Piperacillin, Amikacin, Ampicillin, Gentamicin, Imipenem, Meropenem, Ciprofloxacin.	Bovine Lactoferrin	*E. coli, Citrobacter freundii, Enterobacter aerogene, P. aeruginosa, Staphylococcus epidermidis, S. aureus*	(Al-Mogbel et al. 2021)
Ampicillin, oxytetracycline, streptomycin.	Bovine Lactoferrin	*Listeria, Escherichia coli*.	(García-Borjas et al. 2021)
Amphotericin-B	Lactoferrin	Yeast	(Fernandes, Weeks and Carter 2020)
Levofloxacin, Clarithromycin, Tobramycin.	Bicarinalin	*Helicobacter pylori*	(Iman Zrar Saleh Saleh 2024)
Gentamicin	Temporin A	*S. aureus, P. aeruginosa*	(Paduszynska et al. 2020)
Amoxicillin-clavulanate, Ciprofloxacin, Tobramycin, Imipenem	WLBU2	*Klebsiella pneumoniae, A. baumannii*	(Swedan, Shubair and Almaaytah 2019)
Meropenem, Erythromycin, Amikacin, Ofloxacin	RFR-ChBac3.4(1-14)${\mathrm{N}}{{{\mathrm{H}}}_2}$ (Bactenecin3.4)	*E. coli, K. pneumoniae, A. baumannii, S. aureus, P. aeruginosa*.	(Kopeikin et al. 2020)
Vancomycin	P-113 Derivatives (Nal-P-113, Bip-P-113, Dip-P-113)	*Escherichia coli*, Vancomycin- resistant *Enterococcus faecium* (VRE), vancomycin-intermediate *S. aureus* (VISA), Vancomycin- resistant *S. aureus*.	(Wu et al. 2020)
Colistin (CST)	Citropin 1.1	*P. aeruginosa* PAO1 strain	(Jorge et al. 2017)
Vancomycin, Teicoplanin	Plantaricin NC8 αβ	*Staphylococcus epidermidis*	(Bengtsson et al. 2020)
Penicillin, Ceftazidime, Norfloxacin, Ciprofloxacin.	CM11	*P. aeruginosa, Klebsiella pneumoniae, A. baumannii, S. aureus*,	(Amani et al. 2015)
Azithromycin, Vancomycin	DP7	*P. aeruginosa, A. baumannii, S. aureus, Escherichia coli*.	(Wu et al. 2017)
Rifampicin	hBD-3	*S. aureus*	(Zharkova et al. 2019 )
Rifampicin	HNP-1		
Oxacillin	ChBac3.4		
Gentamicin	hBD-3	*Escherichia coli*	
Rifampicin	ChBac3.4		
Gentamicn	PG-1		
Colistin, Vancomycin, Rifampin	SLAP-S25	MDR *Escherichia coli*	(Song and Zhu 2020)
Colistin	LL-37	*P. aeruginosa*	(Geitani et al. 2019)
Imipenem		MDR *P. aeruginosa*	
Colistin	LL-37	MDR *Escherichia coli*	(Morroni et al. 2021)
Erythromycin	CATH-1	*Escherichia coli, S. aureus, Salmonella enteritidis*	(Lu et al. 2022)

### *In vivo* synergistic interaction of antibiotic-AMPs

While *in vitro* studies provide important proof-of-concept evidence, the evaluation of AMP–antibiotic synergy in *in vivo* models is essential to validate therapeutic potential against MDR pathogens and to optimize dosing strategies for clinical application. Certain synergistic mechanisms, including host immune modulation, pharmacokinetic behavior, and effects on biofilm-associated infections, cannot be adequately assessed under *in vitro* conditions. Consequently, animal models offer critical insights into the efficacy, safety, and translational relevance of these combination therapies.

The *in vivo* safety and performance of AMPs are strongly influenced by peptide concentration and the mode of drug delivery, as many AMPs display dual antimicrobial and immunomodulatory activities (Li et al. 2022). For example, human defensins and the cathelicidin LL-37 function not only as antimicrobial agents but also as regulators of host immune responses, stimulating cytokine production, wound healing, Toll-like receptor signaling, and chemotaxis (Talapko et al. 2022). At physiological or controlled concentrations, these peptides contribute to tissue repair, host defense, and pathogen clearance. However, excessive or uncontrolled AMP exposure may lead to immune dysregulation, enhanced inflammatory responses, and host cell cytotoxicity, thereby limiting systemic application. In particular, elevated concentrations of LL-37 have been associated with inflammation-related tissue damage (Dijksteel et al. 2021).

Additional challenges include limited *in vivo* stability, potential allergenicity, and cytostatic effects toward host cells, all of which further constrain therapeutic applicability (Guryanova and Ovchinnikova ). Therefore, optimization of AMP dosing and the development of suitable drug delivery systems are critical to achieving effective antimicrobial synergy while minimizing immunotoxicity and adverse effects. A comprehensive list of *in vivo* studies investigating AMP–antibiotic synergy is provided in Table 5.

**Table 5.** *In-vivo* studies reporting synergistic interactions between antimicrobial peptides and conventional antibiotics.

Antibiotics	AMPs	Animal Model	Target Pathogen	Synergy mechanism	Ref.
Levofloxacin HCl	PL-5	Mouse infection model	*S. aureus*	PL-5 can destabilize and penetrate biofilm leading to permeability of levofloxacin causing inhibition of DNA gyrase and topoisomerase IV and thus inhibition DNA replication.	(Feng et al. 2015; Fernandez et al. 2021; Taheri-Araghi 2024a)
Penicillin, Ampicillin, Erythromycin	I1WL5W	Mouse infection model	*Staphylococcus epidermidis*	I1WL5W disrupts inner and outer bacterial membrane leading to permeability of antibiotics. Also, this combination inhibits the biofilm increasing the access of antibiotic for intracellular targets.	(Shang et al. 2019; Mhlongo et al. 2023)
Vancomycin	SLAY-P1	*Galleria mellonella*	*Enterococcus*	SLAY-P1 block the transcription of two component vanRS system which is a key regulator resistance to vancomycin thus causing the accumulation of precursor of cell and activity of vancomycin.	(Liu et al. 2020a, 2020b)
Ciprofloxacin, Meropenem, Vancomycin, Gentamicin, Erythromycin	DJK-5, 1018, HHC-10, 1002	Murine sub-cutaneous abscess model	Mixed ESKAPE pathogens (*S. aureus, A. baumannii, C. albicans, E. faecium, Enterococcus cloacae, K. pneumoniae, P. aeruginosa*)	These peptides inhibit the biofilm formation and AMPs 1018 and DJK-5 also disrupts bacterial stress response thus further enhance the efficacy for therapy and ease the intracellular target for antibiotics leading to synergy.	(Pletzer, Mansour and Hancock 2018)
Penicillin, Flucloxacillin, Gentamicin, Neomycin,	Plectasin	Murine neutropenic thigh model, Murine peritoneal infection model	Methicillin-resistant and methicillin- sensitive *S. aureus*	Disruption of cell wall helps entry of neomycin and gentamicin and plectasin (AMP) binds lipid II molecule inhibiting the synthesis of peptidoglycan key component of bacterial cell wall thus making bacteria susceptible to antibiotics.	(Hu et al. 2015; Jekhmane et al. 2024)
Azithromycin, Rifampicin	CEP-136	Murine peritonitis model	*S. aureus, A. baumannii, E. coli, Klebsiella pneumoniae, P. aeruginosa*	CEP-136 disrupt lipopolysaccharide layer of Gram-negative bacteria facilitating the entry of antibiotics and demonstrate low cytotoxicity and hemolytic activity displaying overall synergy.	(Baker et al. 2019; Mood et al. 2021; Wesseling and Martin 2022a)
Gentamicin, Tetracycline	Procaine myeloid antimicrobial peptide 36 (PMAP-36)	Systemic infection mouse model	Procaine extraintestinal pathogenic *Escherichia coli* (ExPEC)	PMAP-36 facilitate by translocating across bacterial cell membrane, binding bacterial DNA increasing the entry of antibiotics and thus complementing the inhibition of bacterial proteins. Also, under electron microscope shrinkage of cell wall and lysis is observed.	(Lu et al. 2022; Tao et al. 2023; Taheri-Araghi 2024b)
Colistin	A3-APO	Murine bacteremia model	*Klebsiella pneumoniae, A. baumannii, Escherichia coli*	Synergy is dependent on the dose and AMP A3-APO target DnaK, the heat shock proteins, thus disrupt the stress response and resistance enzymes leading to bacterial sensitize to colistin.	(Ostorhazi et al. 2011; Otvos Jr. et al. 2018; Xiong et al. 2019)
Ceftazidime	ARV-1502	Melioidosis model		ARV-1502 target DnaK, bacterial chaperone protein thus facilitates in disruption of protein folding and its stress response and involved in immune modulation.	
Ceftriaxone	RN7-IN8	Bacteremia infection model	*Streptococcus pneumonia*	Morphological changes and rapid ATP efflux are caused by RN7-IN8 leading to bacterial membrane and cell wall damages facilitate the mechanism of ceftriaxone.	(Jindal et al. 2015, 2017; Soren et al. 2015)
Oxacillin, Vancomycin, Streptomycin	CSM5-K5	Mouse excisional wound infection model	Methicillin resistant *S. aureus*, Vancomycin-resistant *E. faecalis*, MDR *E. coli*.	The lysine-rich cationic chain of CSM5-K5 electrostatically cluster around and interacts bacterial membrane lipid leading to perturbation and permeability.	(Hou et al. 2017; Thappeta et al. 2019, 2020)
Coumermycin A1, Novobiocin, Erythromycin, Rifampicin, Rifaximin, Vancomycin	D-11	Mouse cutaneous abscess infection model	*Klebsiella pneumoniae, A. baumannii, E. coli, P. aeruginosa*.	D-11 decrease the production ATP, block bacterial efflux pumps and dissolve its proton motive force and bind to bacterial lipopolysaccharides and phospholipid leading to membrane permeability and enhance the activity of combined antibiotics.s	(Cebrián et al. 2021; Li et al. 2021; Wesseling and Martin 2022b)
Rifampicin, Minocycline	Colistin	Neutropenic mouse thigh model	NDM and MCR-1-co- producing *Escherichia coli*	Colistin disrupts bacterial membrane leading to rifampicin causing RNA inhibition and minocycline causes the inhibition of bacterial protein leading to destruction of bacteria.	(Zhou et al. 2014; Yu et al. 2018)
Azithromycin	EC-5, KR-12-a2, L-11, D-11	Murine abscess infection model	*P. aeruginosa*	Peptides impair bacterial electron transport chain causing accumulation of reactive oxygen species and metabolic stress response generation, thus killing the bacteria rapidly. D-11 and azithromycin also block the biofilm formation.	(Lin et al. 2015; Baker et al. 2019; Xia et al. 2021)
Tigecycline	Pexiganan	Murine model of sepsis	*P. aeruginosa* sepsis	Combination of Tigecycline and Pexiganan suppress acquired and intrinsic mechanisms of resistance and facilitate by disruption of membrane and protein synthesis inhibition.	(Cirioni et al. 2019)
Vancomycin	LL-37	MRSA infected mice model	MDR-*S. aureus*	The combination working by blocking the formation of biofilm and help restoring the sensitivity of vancomycin against *Staphylococcus aureus* resistant strain.	(Wu et al. 2021; Han, Wei and Camesano 2022; Lai et al. 2022)
Colistin	Endolysin ElyA1	Murine skin and lung infection model	*A. baumannii, P. aeruginosa*	Biofilms and kill persisters cells which are tolerant to antibiotics are disrupted by endolysin. Additionally, colistin facilitates endolysin to reach and break peptidoglycan layer leading to cell lysis.	(Thummeepak et al. 2016; Blasco et al. 2020; Wojciechowska 2025)
Fosfomycin, Minocycline	Colistin	MDR *A. baumannii* Pneumonia Mouse model	MDR *A. baumannii*	For colistin and fosfomysin combination the synergy is due the production of reactive oxygen species and transcription inhibition of ribosomal protein cause bacterial killing.	(Ku et al. 2019; Nwabor et al. 2021; Zhang et al. 2024)

### Porins as permeability modulators in AMP–antibiotic synergy

Beyond empirical synergy observations, mechanistic determinants of antibiotic entry critically shape combination outcomes. Efficacy of AMP–antibiotic combinations is strongly influenced by the permeability properties of the Gram-negative outer membrane. While AMPs primarily interact with LPS and membrane lipids rather than relying on porin-mediated transport, porins remain critical determinants of antibiotic influx, particularly for hydrophilic compounds such as β-lactams, fluoroquinolones, and carbapenems (Masi et al. 2017, Acosta-Gutiérrez et al. 2018, Vergalli et al. 2020). This mechanistic divergence provides a robust framework to explain how AMPs potentiate antibiotic activity without sharing identical uptake pathways.

Porins such as OmpC, OmpF, OmpK35, OmpK36, and OprD function as selective diffusion channels that regulate periplasmic antibiotic accumulation in Enterobacteriaceae and *P. aeruginosa* (Masi et al. 2017, Vergalli et al. 2020). Alterations in porin expression or structure significantly reduce antibiotic influx and frequently coexist with increased efflux pump activity and enzymatic degradation, collectively shaping MDR phenotypes (Masi et al. 2017, Dam et al. 2018). In the context of AMP–antibiotic combinations, these permeability barriers do not directly impair AMP activity but critically modulate the degree to which antibiotics can exploit AMP-induced membrane perturbations.

Several AMPs have been shown to destabilize the outer membrane by neutralizing surface charges, disrupting LPS packing, or inducing transient membrane defects, thereby lowering the energetic barrier for antibiotic entry (Andersson et al. 2016, Mookherjee et al. 2020). Such perturbations can enhance antibiotic penetration either by facilitating diffusion through residual functional porins or by partially bypassing porin-dependent uptake routes (Mookherjee et al. 2020). Consequently, strains retaining functional porins often display more pronounced synergistic effects *in vitro* and *in vivo*, whereas porin-deficient mutants may exhibit reduced or strain-dependent synergy despite remaining intrinsically susceptible to membrane-active peptides.

Importantly, porin loss rarely occurs in isolation in clinical isolates but instead forms part of a coordinated permeability control system involving porins, efflux pumps, and regulatory stress responses (Masi et al. 2017, Dam et al. 2018). Under these conditions, AMPs may still enhance antibiotic efficacy, but higher peptide concentrations or optimized dosing strategies are often required to overcome compounded permeability barriers. This provides a mechanistic explanation for the variability reported across synergy studies and underscores the importance of considering porin expression profiles when interpreting combination outcomes (Andersson et al. 2016, Lázár et al. 2022).

Recent molecular-level analyses have revealed that outer membrane porins play an indirect yet mechanistically relevant role in shaping AMP interactions with Gram-negative bacteria. Using atomistic molecular dynamics simulations, it has been demonstrated that OmpF induces localized disruption of LPS packing at the protein–lipid interface, generating transient regions of reduced lateral cohesion within the outer membrane. These porin-associated membrane heterogeneities promote the preferential accumulation and stabilization of short, cationic AMPs at the LPS surface, even in the absence of direct pore translocation. In particular, the presence of OmpF was shown to expose negatively charged phosphate groups within the inner core oligosaccharides, lowering the energetic barrier for peptide insertion and membrane perturbation. These findings support a model in which porins do not function as obligatory entry routes for AMPs but instead act as structural organizers that locally weaken the LPS barrier, thereby facilitating peptide access and activity (Di Somma et al. 2022).

Complementary experimental and computational evidence further indicates that porin-mediated modulation of outer membrane organization has direct consequences for antibiotic susceptibility and AMP–antibiotic synergy. Recent integrative studies combining membrane permeability assays, microscopy, and computational modeling have shown that AMP-induced destabilization of the outer membrane enhances antibiotic penetration primarily in strains retaining residual porin functionality. Rather than compensating entirely for porin loss, AMPs lower the permeability threshold required for antibiotic influx, thereby amplifying the efficacy of hydrophilic antibiotics, whose uptake remains partially porin-dependent. Importantly, this framework explains why synergistic interactions are often attenuated—but not abolished—in porin-deficient mutants, and why strain-dependent variability is frequently observed in combination studies. By linking porin expression status, membrane architecture and peptide-induced perturbations, these findings underscore the need to consider outer membrane organization as a dynamic and cooperative determinant of AMP–antibiotic synergy (Necula et al. 2023).

Overall, porins should not be regarded as primary resistance determinants against AMPs but rather as key modulators of antibiotic access, whose functional status shapes the outcome of AMP–antibiotic synergy (Mookherjee et al. 2020, Lázár et al. 2022). Integrating outer membrane permeability characteristics into experimental design and translational strategies may therefore improve the predictability and clinical relevance of peptide-based combination therapies against MDR Gram-negative pathogens.

## Challenges to the clinical applications of antibiotic–AMPs combination therapy

Despite their therapeutic promise, the clinical translation of AMPs—either alone or in combination with antibiotics—remains constrained by biological, pharmacological, and regulatory barriers. Importantly, synergy is not a fixed property: it is shaped by pathogen-specific envelope states, adaptive responses under peptide exposure, and host microenvironmental conditions that are difficult to reproduce in standard *in vitro* assays.

### Biological and mechanistic constraints limiting synergy

Synergy between antibiotics and AMPs typically relies on complementary mechanisms, most commonly AMP-driven perturbation of the bacterial envelope that increases antibiotic access to periplasmic or intracellular targets. However, this cooperation can be disrupted by resistance-associated adaptations or stress responses that remodel the cell surface, alter permeability, and promote cross-resistance, thereby reducing the predictability and durability of AMP–antibiotic combinations.

#### Membrane remodeling and loss of AMP–antibiotic synergy

In Gram-negative bacteria, envelope remodeling can directly compromise synergy by reducing AMP binding and limiting downstream antibiotic entry. For example, *mcr-1*-mediated phosphoethanolamine modification of lipid A impairs polymyxin binding and can blunt permeabilization-dependent potentiation of partner antibiotics (Liu et al. 2016). In Gram-positive bacteria, the aps regulatory system in MRSA senses host-derived peptides and triggers protective responses—including surface charge alteration and efflux—thereby weakening AMP activity and diminishing synergy (Andersson et al. 2016, Olaitan et al. 2016). Similarly, LPS modification via addition of 4-amino-4-deoxy-l-arabinose in Gram-negative species, or incorporation of lysyl-phosphatidylglycerol in *S. aureus*, reduces the net negative surface charge and decreases binding affinity of cationic AMPs, ultimately lowering antibiotic uptake in combinations that depend on peptide-driven permeabilization (Nawrocki et al. 2014).

Adaptive responses can be amplified by repeated or prolonged exposure. *In vitro* studies show that sublethal exposure to LL-37 or defensins can reduce susceptibility in *S. aureus*, and clinical *P. aeruginosa* isolates from cystic fibrosis patients frequently display decreased sensitivity to HDPs, consistent with adaptation to sustained selective pressure (Camus et al. 2021). A major concern is cross-resistance between synthetic AMPs and HDPs: envelope modifications that reduce susceptibility to therapeutic AMPs can simultaneously decrease defensin activity, potentially undermining innate immune effectiveness (Nawrocki et al. 2014). Two-component systems such as PhoPQ and PmrAB contribute to these adaptive states by regulating efflux and membrane modification programs (altering charge and/or permeability). Disruption of PhoPQ increases AMP susceptibility in *S. enterica*, whereas activation of PmrAB-associated remodeling can yield a more rigid and less permeable membrane phenotype (May and Groisman 2013, Pranantyo et al. 2022). Although AMPs are often described as less prone to resistance development than conventional antibiotics, combination therapy may still select for envelope adaptations and cross-resistance pathways that erode synergy over time.

#### Subversion of host-derived AMPs

Beyond canonical resistance mechanisms, some pathogens can coopt host-derived AMPs in ways that enhance survival or virulence, complicating the therapeutic deployment of AMP-based strategies. *Shigella flexneri* provides a well-studied example: lipid A acylation and dephosphorylation reduce outer membrane negative charge, decreasing the binding affinity for cationic AMPs. In parallel, host-derived peptides such as LL-37 and intestinal α-defensin 5 can be exploited to promote epithelial adhesion by overcoming electrostatic repulsion at the host–pathogen interface. This phenomenon reframes AMPs not only as antimicrobial effectors but also as context-dependent modulators of infection dynamics (Xu et al. 2018, Eilers et al. 2010).

Similarly, *Legionella pneumophila* can hijack host lysosomal trafficking networks and manipulate Rab7-dependent maturation to remodel phagosomes into replication-permissive vacuoles (Xu et al. , Li et al. 2024a). Although this is not “AMP resistance” in the strict pharmacological sense, it illustrates how intracellular pathogens can remodel host compartments to persist in AMP-rich environments, representing a host–pathogen interaction constraint that may limit therapeutic predictability. Together, these examples highlight that AMP biology can intersect with virulence strategies, and therefore resistance frameworks should account for AMP subversion as a translational barrier, not only as a microbial evasion mechanism.

### Pharmacokinetic, pharmacodynamic, and toxicity-related barriers

From a translational perspective, pharmacological constraints represent a major bottleneck for the clinical application of AMP–antibiotic combination therapies. Many AMPs exhibit nonlinear pharmacokinetics and lack validated PK/PD indices, complicating dose selection and exposure optimization. In addition, limited selectivity can lead to hemolytic or cytotoxic effects at concentrations required for antimicrobial efficacy; for example, the plant-derived thionin CaThi showed potent antifungal activity but induced corneal toxicity in human models (Taveira et al. 2017). AMP activity may also be reduced in physiological environments: high salt can weaken electrostatic interactions required for activity, and host proteases can rapidly degrade peptides, reducing half-life, bioavailability, and consequently synergistic potential with antibiotics (Starr and Wimley 2017, Li et al. 2021, Lai et al. 2022, Xian et al. 2022).

Poor membrane permeability and oral bioavailability remain common obstacles for the clinical deployment of AMP–antibiotic combinations. Classical drug-likeness criteria (e.g. Lipinski-type constraints) are often violated by peptides, contributing to limited absorption and distribution (Lipinski et al. 2012). Alternative descriptors such as exposed polar surface area have been proposed, yet many cyclic peptides still show unsatisfactory permeability in practice, despite documented exceptions (Rezai et al. 2006, White et al. 2011, Hill et al. 2014, Fouché et al. 2016). Peptide half-life is further limited by enzymatic degradation and renal clearance; plasma and tissue proteases can inactivate peptides, and rapid filtration contributes to short systemic exposure (Vlieghe et al. 2010).

Crucially, *in vitro* synergy does not reliably predict *in vivo* benefit; for instance, meropenem–colistin combinations may show *in vitro* synergy against *A. baumannii* yet fail to improve outcomes *in vivo*, underscoring the difficulty of reproducing physiological conditions (pH, salts, nutrients, and osmotic pressure) in standard assays (Paul et al. 2018, Liu et al. 2020). Antagonism has also been observed for some AMP–antibiotic pairs, driven by direct molecular interference, species-specific differences, or unfavorable pairing of mechanisms (e.g. competitive binding or functional redundancy), as reported for certain combinations tested against *S. aureus* (Oshiro et al. 2008, Lalouckova et al. 2021). Finally, polymyxins—frequently used in AMP–antibiotic synergy studies—remain clinically limited by nephrotoxicity, linked to renal tubular injury pathways involving oxidative stress, apoptosis, and autophagy (Zavascki and Nation 2017, XIAO et al. 2022).

### Regulatory and formulation hurdles

Regulatory barriers further slow translation, as multiple AMP candidates (e.g. iseganan, pexiganan, and neuprex) failed in late-phase trials due to insufficient efficacy compared with existing standards of care, a minimum requirement for FDA approval (Koo and Seo 2019, Dijksteel et al. 2021). Other peptides are restricted to topical use because of systemic toxicity (e.g. gramicidin) (Gai et al. 2019), while additional candidates require dose adjustments due to nephrotoxicity (e.g. mucomycin) (Li et al. ). Mammalian AMPs—often cathelicidins and defensins—can display cytotoxicity, hemolysis, and proinflammatory effects *in vitro* and *in vivo*, limiting systemic administration at therapeutically relevant exposures (e.g. GF-17/LL-37 fragments, LL-37, indolicidin, Dhvar-5, protegrin-1, HNP-1/4, and hBD-2/3) (Zharkova et al. 2019). Consequently, most AMP formulations remain confined to topical or intravenous routes, limiting compliance and flexibility; oral development has often shown low efficacy and narrow therapeutic windows. (Zheng et al. 2025).

Advanced delivery approaches, including nanocarrier-based systems such as PLGA–PEG nanoparticles functionalized with AMPs, may improve stability, bioavailability, and targeting, but they introduce regulatory uncertainty regarding classification (biopharmaceutical versus medical device), which can delay approval pathways (Zheng et al. ). Overall, these barriers underline the persistent gap between promising preclinical synergy and successful clinical deployment.

## Strategies to improve AMP–antibiotic combination therapy

To overcome these obstacles, several complementary strategies have been proposed. Peptide modification remains one of the most direct approaches, aimed at improving stability, specificity, and reducing toxicity. Strategies such as peptidomimetics, cyclization, substitution, and dimerization have yielded shorter and more stable analogues with reduced production costs, exemplified by clinical candidates such as EA-230 and LTX-109 (Midura-Nowaczek and Markowska 2014, van Groenendael et al. 2018). Truncated or ultrashort peptides are also being explored for economic synthesis, while nanoscale delivery systems—including liposomes, niosomes, and polymeric nanoparticles—are employed to enhance bioavailability, solubility, and therapeutic index (Mishra et al. 2017). Innovative formulations such as colon-specific delivery of polymyxin E have shown success in bypassing gastrointestinal degradation and improving topical efficacy in burn wound models (Zhu et al. 2017).

Precision-based approaches are equally critical. Pathogen-specific combination therapy leverages knowledge of pathogen resistance mechanisms and infection site environments to design AMP–antibiotic pairings with complementary modes of action (Zhu et al. 2022). A more refined concept is that of specifically targeted AMPs, which combine pathogen-binding motifs with killing domains to achieve selective elimination of pathogens without disrupting commensal flora. The synthetic peptide C16G2, currently under clinical evaluation for *Streptococcus mutans*, exemplifies this design by demonstrating strong efficacy and safety through its dual-terminal structure (He et al. 2010, Kaplan et al. 2011). In parallel, computational modeling, high-throughput screening, and bioinformatics resources (e.g. AMP databases) are increasingly applied to predict synergistic AMP–antibiotic pairs and accelerate discovery pipelines (Mookherjee et al. , Wang et al. 2016, Torres et al. 2021, Hao et al. 2022). Together, these strategies suggest that with targeted design, optimized delivery, and integrative computational support, AMP–antibiotic combinations can be transformed from promising *in vitro* findings into clinically relevant therapies capable of addressing AMR.

## Future research directions

The rapid emergence of MDR pathogens underscores the urgent need for novel antimicrobial strategies, with AMP–antibiotic combinations representing one of the most promising avenues. These combinations exploit complementary mechanisms to restore antibiotic efficacy, broaden activity spectra, and reduce resistance emergence. Considerable progress has been made in identifying synergistic AMP–antibiotic pairs with activity against multidrug-resistant pathogens. However, several areas require further development before these approaches can achieve robust clinical application. A first priority is the deeper mechanistic understanding and optimization of synergy: while *in vitro* studies provide valuable insights into bacterial killing and resistance suppression, translation into *in vivo* models and human clinical trials remains challenging, demanding extensive pharmacokinetic and safety evaluations (Seyhan 2019, Dijksteel et al. 2021, Talapko et al. 2022).

In parallel, the rational design of delivery systems and formulations tailored to pathogen resistance profiles is crucial. Computational models, high-throughput screening, and AMP databases already facilitate the identification of effective AMP–antibiotic pairs (Zhang and Yang 2022). In this context, molecular dynamics simulations are increasingly employed to predict peptide–membrane interactions, antibiotic permeability, and synergistic mechanisms at atomic resolution, providing mechanistic validation that complements experimental observations, particularly when coupled to advanced structural platforms (Roque-Borda et al. 2025b, Scharbert and Strodel 2025). In parallel, liposomes, micelles, nanocarriers, and AMP modifications are being explored to enhance stability, reduce cytotoxicity, and provide site-specific delivery (Tan et al. 2021, Primo et al. 2024). Another critical focus is the management of biofilm-associated and persistent infections, which remain major clinical challenges. Although some combinations show promise, AMPs often fail to eradicate biofilms and persister populations effectively, necessitating further innovation; AMP-loaded hydrogels or coatings for medical devices are emerging as potential solutions for chronic wounds and implant-related infections (Zhu et al. 2017, Kanaujia et al. 2024).

Beyond classical definitions of synergy based on additive or potentiating effects, an emerging conceptual shift frames AMP–antibiotic combinations as components of multitarget antimicrobial systems. In this framework, therapeutic efficacy arises not solely from enhanced bacterial killing but from the simultaneous disruption of multiple, functionally distinct bacterial processes, including membrane integrity, intracellular metabolism, stress response pathways, and resistance determinants. Importantly, many AMP–antibiotic pairs also exhibit dual-action behavior, in which antimicrobial activity is coupled to immunomodulatory or antivirulence effects, further broadening the functional impact of treatment. By distributing selective pressure across independent targets and biological layers, such multitarget and dual-action strategies may reduce the likelihood of resistance emergence compared with single-target therapies or conventional combinations (Roque-Borda et al. 2025c, Polinário et al. 2023). This perspective aligns with recent efforts to design AMP–antibiotic pairs that act cooperatively at the membrane, periplasmic, and cytoplasmic levels, thereby increasing the evolutionary barrier to resistance while preserving therapeutic flexibility across diverse bacterial phenotypes.

Advances in next-generation sequencing and rapid diagnostics also open opportunities for pathogen-specific therapies, where tailored combinations based on genomic and phenotypic resistance profiles could improve therapeutic success and minimize microbiome disruption (Sarma et al. 2018, Zhu et al. 2022). Finally, improving AMP robustness *in vivo* remains essential, as peptides are often unstable under physiological conditions characterized by high salt concentrations and protease activity. Future efforts should therefore focus on engineering structurally stable AMPs, ideally integrated with nanotechnology-based delivery systems, to maximize stability, bioavailability, and therapeutic efficacy (Tan et al. 2021). In this context, recent advances in peptide chemistry and computational design are shifting AMPs from empirically optimized sequences toward purpose-driven, structurally programmed molecules, enabling improved stability, selectivity, and functional integration in combination therapies with conventional antibiotics (Roque-Borda et al. 2025a).

## Conclusion

AMR represents a critical and escalating global health challenge that continues to erode the effectiveness of conventional antibiotics. AMPs offer distinct mechanistic advantages, including membrane-targeting activity and immunomodulatory functions; however, their clinical translation remains constrained by pharmacokinetic limitations, instability, toxicity, and context-dependent efficacy. AMP–antibiotic combination therapy has emerged as a compelling strategy to overcome these limitations, yet this review highlights that synergistic interactions are neither universal nor guaranteed. Instead, synergy is shaped by a complex interplay of factors, including bacterial envelope architecture, porin-mediated permeability, resistance-associated adaptations, host immune modulation, and pharmacological compatibility between agents. Importantly, strong *in vitro* synergy does not necessarily translate into *in vivo* benefit, underscoring the need for mechanistically informed experimental design and appropriate validation models. By moving beyond descriptive synergy toward a mechanistic and translational framework, AMP–antibiotic combinations may contribute meaningfully to addressing MDR bacterial infections.

## Supplementary Material

xtag003_Supplemental_File

## Data Availability

There are no datasets presented in this paper.
