# Supplementary material for: Enhanced antibacterial activity of antimicrobial peptide—antibiotic combinations against multidrug-resistant bacteria
Source: FEMS Microbes. 2026 Feb 4;7:xtag003. doi: 10.1093/femsmc/xtag003 (PMC12947588; doi:10.1093/femsmc/xtag003)
Supplement: xtag003_Supplemental_File [file xtag003_supplemental_file.docx]

**Reviews of manuscript FEMSMC-2025-042**

**Reviewer 1**

**Recommendation: Major Revision**

The review, titled "Overview of Antimicrobial Peptide and Antibiotic Synergy Against Multidrug Resistant Bacteria," presents an exploration of the mechanisms of action of antimicrobial peptides (AMPs) and antibiotics, bacterial resistance strategies against AMPs, and the translational barriers of these compounds. While the subject matter is pertinent, the manuscript's current structure leans towards a narrative review, potentially diluting its focus on the central theme of synergy, as explicitly stated in the title.

1. The review's primary focus should be rigorously maintained on AMP-antibiotic synergy. It is strongly recommended that the manuscript be restructured to emphasize Topic 4 (presumably dedicated to synergy) with a more detailed and analytical discussion.

2. Topics 2 and 3 (likely generic mechanisms of action and resistance) should be either deleted or significantly curtailed and integrated into an introductory section. If kept, their content should be directly linked to how these aspects influence or are relevant to the synergistic action.

Specific Content Revisions

1. Definition of Antimicrobial Peptides (AMPs) (Line 71): The current definition, "Antimicrobial peptides (AMPs) represent such an alternative. These cationic, amphipathic molecules...", is overly restrictive and outdated. A broader, more general description of AMPs as a diverse class is necessary. Acknowledge that while many well-studied AMPs are cationic and amphipathic, a subset of AMPs possess a negative net charge (anionic AMPs) and function through distinct mechanisms.

2. Confusing Phrase (Lines 126–128): The phrase is currently confusing and lacks clarity. It must be rephrased for conciseness and scientific precision, and its underlying scientific concept should be expanded upon.

3. Figure 1 Legend: The legend is summarized and requires completion to fully describe the figure's contents. Furthermore, the figure's placement and/or sequence must be re-ordered to ensure alignment with its first mention in the main text.

4. The structural separation into Section 2. "Antimicrobial Peptides and Antibiotics" and Section 3. "Antimicrobial Peptides" is not practical and creates redundancy. These sections should be merged or completely reorganized under the principle of thematic focus. I suggest that it should be deleted.

5. The descriptions of AMPs in Lines 95 and 241 are repetitive. Consolidate the definitional and mechanistic description into a single, cohesive introductory section.

6. The statement that electrostatic interaction "allow selective interaction... leading to membrane destabilization and cell death" is an oversimplification. Clarify that the initial electrostatic interaction (due to the net positive charge of the AMP and negative charge of the bacterial membrane) is the basis for selectivity, but the subsequent mechanism of action and ultimate cell death are attributed to the formation of specific structures such as pores (e.g., barrel-stave or toroidal models) or a detergent-like effect (carpet mechanism).

7. Classification of AMPs (Item 3.1): The section titled "3.1. Classification of AMPs" currently fails to provide a classification scheme.

8. Data Visualization (Figures 2 and 3): The suggestion to reformat Figures 2 and 3 into bar graphs should be considered if the data represents distinct categories, comparisons, or discrete quantitative outcomes.

9. Functional Classification (Item 3.1.1.1): The "Functional Classification" section must be inclusive of all known functional activities. The omission of anticancer peptides is a gap, as many AMPs exhibit dual-functionality (antimicrobial and anticancer), a property driven by similar membrane-targeting selectivity against the distinct surface charge of cancer cells. This omission should be addressed for completeness in a functional context.

**Reviewer 2: Daniel Propheter, The University of Texas Southwestern Medical Center**

**Recommendation: Major Revision**

This review is supposed to provide an overview of the synergism of antimicrobial peptides (AMPs) and antibiotics in combating multidrug resistant bacteria. This is a topic of significance as the number of MDR infections will continue to outpace the development of new drugs. While a promising topic, I believe the authors can make this review much more appealing.

My suggestions to improve this manuscript are as follows:

1) Section 2: I believe this section is far too long; the target audience is very likely to be well-versed in small molecule antibiotics. I would suggest the authors reduce this entire section and perhaps include a table to help summarize.

2) Section 3.1.1.1: I think the authors should remove sections C, D, and E since these sections have nothing to do with the antimicrobial functions of AMPs and don't add anything of value to the topic at hand.

3) Sections 3.2 and 3.3: As currently written, I think these sections are not as helpful as they could be. I would recommend that the authors re-construct this section by looking at AMPs from different sources (e.g. microbial, plant, animal) and their different mechanisms-of-action (MOAs). Then conversely, do the same in section 3.3 on microbial resistance mechanisms.

4) Tables 1 and 2: These tables, although full of information, are not informative on the topic of antibiotic and AMP synergism. The simply list the antibiotic, AMP, and target pathogen. I think it would help the manuscript by expanding the number of tables and content. First, I don't find Table 1 to be important considering it is in vitro data. The in vivo data (Table 2), should be expanded upon. I would suggest the authors focus on MOA between the antibiotic and AMP. It would be very useful for the reader to know whether an antibiotic and an AMP with the same MOA is more effective than a set with different MOAs.

5) Section 5, paragraph starting at line 616: I think the authors should highlight that these safety issues are limited to microbial AMPs that they listed. Is there safety data available for mammalian AMPs?

In summary, this is a topic of great importance and I think the authors should focus more of their efforts on AMPs and potential synergism. And perhaps make some suggestions on what antibiotic-AMP combinations may prove to be more useful than other combinations.

**Reviewer 3: Sean Van Helden, Wayne State University**

**Recommendation: Major Revision**

This manuscript by Talha and Roque-Borda is a comprehensive and digestible review of antimicrobial peptides (AMPs) that provides scientists and clinicians with an overview of their characteristics, therapeutic potential, and issues relating to clinical translation. The authors reviewed the issue of multidrug-resistant bacteria, antibiotic resistance mechanisms, AMP classification, AMP mechanisms of action and bacterial resistance, and clinical translation. The following issues should be addressed prior to publication:

Major comments:

1. There are many instances of statements made in the manuscript where a citation is required but is lacking (i.e. lines 8-, 206, 212, 238, 293, 312, 422, 470-480). Please ensure that citations are provided for all statements of fact made in this review.

2. Please comment on clinical safety of AMP administration in vivo. Particularly the interplay between the antimicrobial activity of AMPs and host immune regulation/dysregulation in the context of therapeutic administration of AMPs.

3. It appears that porin loss has an impact of AMP therapeutic efficacy (PMIDs: 36768325, 36324521). Consider adding a subsection under section 3.3 commenting on the impact of porin loss on AMP penetration and efficacy.

4. Line 599: please comment on the short half-lives of AMPs and how it impacts clinical translation as therapeutic agents.

Minor comments:

1. Please ensure all genus and species are italicized consistently throughout the manuscript, including yeasts and viruses.

2. Line 55: conventional antibiotics fail in biofilm due to additional mechanisms other than poor penetration, including differences in bacterial metabolic activity (limits antibiotics targeting cell replication), drug tolerance, etc. Please add these mechanisms to this statement.

3. Line 62: Group is now referred to as Enterobacterales. Please amend.

4. Lines 145-146: Co-trimoxazole is not recommended as first line therapy in treating respiratory tract infections given its lack of effective streptococcus activity. Please amend or remove this statement.

5. Line 154: Consider listing the protein synthesis inhibitor classes (macrolides, aminoglycosides, oxazolidinones, tetracyclines)

6. Line 187: Please change to correct citation style.

7. Line 194: Please define abbreviation

8. Line 201: would add in PBP mutations, as these are responsible for resistance to novel BLBLIs

9. Lines 297-299: This sentence does not make sense. Please revise.

10. Lines 350-357: There are no capital letters in this section. Please revise.

11. Line 418: Please briefly elaborate on the mechanism by which AMPs are able to cross the outer membrane of Gram-negative organisms.

12. Line 470: amend section title to something like “bacterial resistance to AMPs” to more accurately reflect the section’s contents.

13. Line 622: While vancomycin is technically an AMP, most clinicians and researchers would consider it an antibiotic and distinct from other AMPs such as LL37. Therefore, its inclusion in this line is confusion and should be revised or removed. Consider inserting other instances of attempted oral formulation development or briefly explaining why vancomycin is an effective representation of the barriers facing AMPs with regard to oral formulation.

**Associate Editor Recommendation: Major Revision**

Thank you for your patience while we secured reviews for your manuscript. The work has now been reviewed by three experts in the field. All are in agreement that the topic is important and that it is a timely review. However, there are several recommendations to improve the manuscript. This includes more critically focusing the majority of the review topic on the subject of AMP-antibiotic synergy which is lacking. There are also numerous suggestions to shorten overly lengthy sections, or sections that provide basic background, that stray from the main theme of the review. I agree with the reviewers that an overhaul of this review is needed and that much more depth and emphasis needs to be paid to the topic of AMP-antibiotic synergy. Finally, I feel that moving forward, in the title you should remove the reference to "Overview" and simply have the title be "Antimicrobial Peptide and Antibiotic Synergy Against Multidrug Resistant Bacteria"

**EIC Decision: Major Revision**

**Author's Response to Decision Letter for (FEMSMC-2025-042)**

Response to the Associate Editor and Reviewers

Associate Editor

Comments to the Author:

Thank you for your patience while we secured reviews for your manuscript. The work has now been reviewed by three experts in the field. All are in agreement that the topic is important and that it is a timely review. However, there are several recommendations to improve the manuscript. This includes more critically focusing the majority of the review topic on the subject of AMP-antibiotic synergy which is lacking. There are also numerous suggestions to shorten overly lengthy sections, or sections that provide basic background, that stray from the main theme of the review. I agree with the reviewers that an overhaul of this review is needed and that much more depth and emphasis needs to be paid to the topic of AMP-antibiotic synergy. Finally, I feel that moving forward, in the title you should remove the reference to "Overview" and simply have the title be "Antimicrobial Peptide and Antibiotic Synergy Against Multidrug Resistant Bacteria".

Response: We thank the Associate Editor for this clear and constructive assessment. In response, the manuscript has undergone a substantial overhaul to refocus the review on AMP–antibiotic synergy as the central theme. Overly long background sections have been shortened or restructured to explicitly link mechanistic and resistance-related content to synergistic outcomes. In addition, the manuscript title has been revised to remove the term “Overview” and to emphasize AMP–antibiotic synergy, in accordance with the recommendation.

Reviewer 1

Comments to the Author:

The review, titled "Overview of Antimicrobial Peptide and Antibiotic Synergy Against Multidrug Resistant Bacteria," presents an exploration of the mechanisms of action of antimicrobial peptides (AMPs) and antibiotics, bacterial resistance strategies against AMPs, and the translational barriers of these compounds. While the subject matter is pertinent, the manuscript's current structure leans towards a narrative review, potentially diluting its focus on the central theme of synergy, as explicitly stated in the title.

Response: We agree with this assessment. The manuscript has been restructured to reduce its narrative character and to strengthen the analytical focus on AMP–antibiotic synergy. Background content has been condensed, and new sections explicitly addressing in vitro synergy, in vivo validation, and mechanistic determinants of synergy have been added or expanded.

1. The review's primary focus should be rigorously maintained on AMP-antibiotic synergy. It is strongly recommended that the manuscript be restructured to emphasize Topic 4 (presumably dedicated to synergy) with a more detailed and analytical discussion.

Response: The revised manuscript places increased emphasis on AMP–antibiotic synergy, particularly through expanded discussion of in vitro and in vivo synergistic interactions and a restructured section addressing translational challenges. Analytical depth has been added to discuss how mechanistic factors influence synergistic outcomes.

2. Topics 2 and 3 (likely generic mechanisms of action and resistance) should be either deleted or significantly curtailed and integrated into an introductory section. If kept, their content should be directly linked to how these aspects influence or are relevant to the synergistic action.

Response: Topics 2 and 3 have been significantly curtailed and reorganized. Content has been retained only where it directly informs AMP–antibiotic synergy, such as mechanisms affecting permeability, resistance-associated loss of synergy, or modulation of antibiotic uptake.

Specific Content Revisions

1. Definition of Antimicrobial Peptides (AMPs) (Line 71): The current definition, "Antimicrobial peptides (AMPs) represent such an alternative. These cationic, amphipathic molecules...", is overly restrictive and outdated. A broader, more general description of AMPs as a diverse class is necessary. Acknowledge that while many well-studied AMPs are cationic and amphipathic, a subset of AMPs possess a negative net charge (anionic AMPs) and function through distinct mechanisms.

Response: The definition of AMPs has been revised to reflect their chemical and functional diversity. The revised text explicitly acknowledges the existence of anionic AMPs and their distinct mechanisms of action.

2. Confusing Phrase (Lines 126–128): The phrase is currently confusing and lacks clarity. It must be rephrased for conciseness and scientific precision, and its underlying scientific concept should be expanded upon.

Response: This phrase has been rephrased to improve clarity and precision, and the underlying scientific concept has been expanded to better convey its mechanistic significance.

3. Figure 1 Legend: The legend is summarized and requires completion to fully describe the figure's contents. Furthermore, the figure's placement and/or sequence must be re-ordered to ensure alignment with its first mention in the main text.

Response: The Figure 1 legend has been expanded to fully describe the figure contents, and the figure placement has been adjusted to align with its first mention in the text.

4. The structural separation into Section 2. "Antimicrobial Peptides and Antibiotics" and Section 3. "Antimicrobial Peptides" is not practical and creates redundancy. These sections should be merged or completely reorganized under the principle of thematic focus. I suggest that it should be deleted.

Response: These sections have been reorganized to eliminate redundancy and to follow a thematic structure centered on AMP–antibiotic synergy.

5. The descriptions of AMPs in Lines 95 and 241 are repetitive. Consolidate the definitional and mechanistic description into a single, cohesive introductory section.

Response: Repetitive descriptions have been consolidated into a single introductory section to avoid redundancy.

6. The statement that electrostatic interaction "allow selective interaction... leading to membrane destabilization and cell death" is an oversimplification. Clarify that the initial electrostatic interaction (due to the net positive charge of the AMP and negative charge of the bacterial membrane) is the basis for selectivity, but the subsequent mechanism of action and ultimate cell death are attributed to the formation of specific structures such as pores (e.g., barrel-stave or toroidal models) or a detergent-like effect (carpet mechanism).

Response: This section has been revised to clarify the distinction between initial electrostatic interactions and downstream membrane-disruptive mechanisms, including pore formation and detergent-like effects.

7. Classification of AMPs (Item 3.1): The section titled "3.1. Classification of AMPs" currently fails to provide a classification scheme.

Response: Non-essential classification content has been streamlined or removed to maintain focus on AMP–antibiotic synergy.

8. Data Visualization (Figures 2 and 3): The suggestion to reformat Figures 2 and 3 into bar graphs should be considered if the data represents distinct categories, comparisons, or discrete quantitative outcomes.

Response: This suggestion has been considered, and figure presentation has been revised where appropriate to improve clarity.

9. Functional Classification (Item 3.1.1.1): The "Functional Classification" section must be inclusive of all known functional activities. The omission of anticancer peptides is a gap, as many AMPs exhibit dual-functionality (antimicrobial and anticancer), a property driven by similar membrane-targeting selectivity against the distinct surface charge of cancer cells. This omission should be addressed for completeness in a functional context.

Response: Sections not directly relevant to antimicrobial function and AMP–antibiotic synergy have been removed or condensed to preserve thematic focus.

Reviewer 2

Comments to the Author:

This review is supposed to provide an overview of the synergism of antimicrobial peptides (AMPs) and antibiotics in combating multidrug resistant bacteria...

Response: We thank the reviewer for these constructive suggestions and have revised the manuscript accordingly to improve focus and clarity.

1) Section 2: I believe this section is far too long; the target audience is very likely to be well-versed in small molecule antibiotics. I would suggest the authors reduce this entire section and perhaps include a table to help summarize.

Response: Section 2 has been substantially shortened, retaining only content directly relevant to AMP–antibiotic synergy.

2) Section 3.1.1.1: I think the authors should remove sections C, D, and E since these sections have nothing to do with the antimicrobial functions of AMPs and don't add anything of value to the topic at hand.

Response: Sections C, D, and E have been removed, as they do not contribute to the discussion of antimicrobial function or synergy.

3) Sections 3.2 and 3.3: As currently written, I think these sections are not as helpful as they could be. I would recommend that the authors re-construct this section by looking at AMPs from different sources (e.g. microbial, plant, animal) and their different mechanisms-of-action (MOAs). Then conversely, do the same in section 3.3 on microbial resistance mechanisms.

Response: Sections 3.2 and 3.3 have been reconstructed to focus on in vivo synergy, immune modulation, and mechanistic determinants of synergy, including permeability and resistance mechanisms.

4) Tables 1 and 2: These tables, although full of information, are not informative on the topic of antibiotic and AMP synergism. The simply list the antibiotic, AMP, and target pathogen. I think it would help the manuscript by expanding the number of tables and content. First, I don't find Table 1 to be important considering it is in vitro data. The in vivo data (Table 2), should be expanded upon. I would suggest the authors focus on MOA between the antibiotic and AMP. It would be very useful for the reader to know whether an antibiotic and an AMP with the same MOA is more effective than a set with different MOAs.

Response: The accompanying text has been expanded to critically discuss mechanisms of action underlying AMP–antibiotic synergy.

5) Section 5, paragraph starting at line 616: I think the authors should highlight that these safety issues are limited to microbial AMPs that they listed. Is there safety data available for mammalian AMPs?

Response: The revised manuscript clarifies safety considerations and discusses available data on mammalian AMPs where relevant.

Reviewer 3

Major comments

1. There are many instances of statements made in the manuscript where a citation is required but is lacking (i.e. lines 8-, 206, 212, 238, 293, 312, 422, 470-480). Please ensure that citations are provided for all statements of fact made in this review.

Response: The manuscript has been carefully reviewed to ensure that all factual statements are appropriately supported by citations.

2. Please comment on clinical safety of AMP administration in vivo. Particularly the interplay between the antimicrobial activity of AMPs and host immune regulation/dysregulation in the context of therapeutic administration of AMPs.

Response: This issue is addressed in Section 3.2, which discusses immune modulation, concentration-dependent effects, and safety considerations.

3. It appears that porin loss has an impact of AMP therapeutic efficacy (PMIDs: 36768325, 36324521). Consider adding a subsection under section 3.3 commenting on the impact of porin loss on AMP penetration and efficacy.

Response: A dedicated subsection has been added discussing porin loss and its impact on AMP penetration and AMP–antibiotic synergy.

4. Line 599: please comment on the short half-lives of AMPs and how it impacts clinical translation as therapeutic agents.

Response: This issue is addressed in the section discussing translational challenges and pharmacokinetic limitations.

Minor comments

1. Please ensure all genus and species are italicized consistently throughout the manuscript, including yeasts and viruses.

Response: This has been corrected. All genus and species names, including bacteria, yeasts, and viruses, have been italicized consistently throughout the manuscript.

2. Line 55: conventional antibiotics fail in biofilm due to additional mechanisms other than poor penetration, including differences in bacterial metabolic activity (limits antibiotics targeting cell replication), drug tolerance, etc. Please add these mechanisms to this statement.

Response: This statement has been revised to include additional biofilm-associated mechanisms contributing to antibiotic failure, including reduced metabolic activity, drug tolerance, and phenotypic heterogeneity.

3. Line 62: Group is now referred to as Enterobacterales. Please amend.

Response: This has been corrected. The term Enterobacteriaceae has been replaced with Enterobacterales.

4. Lines 145–146: Co-trimoxazole is not recommended as first line therapy in treating respiratory tract infections given its lack of effective streptococcus activity. Please amend or remove this statement.

Response: This statement has been amended to avoid misrepresentation of current clinical recommendations. The reference to co-trimoxazole as first-line therapy for respiratory tract infections has been removed.

5. Line 154: Consider listing the protein synthesis inhibitor classes (macrolides, aminoglycosides, oxazolidinones, tetracyclines).

Response: This suggestion has been addressed. The relevant protein synthesis inhibitor classes have been explicitly listed in the revised text.

6. Line 187: Please change to correct citation style.

Response: The citation style at this location has been corrected to conform with the journal’s formatting guidelines.

7. Line 194: Please define abbreviation.

Response: The abbreviation has now been defined at its first occurrence in the text.

8. Line 201: would add in PBP mutations, as these are responsible for resistance to novel BLBLIs.

Response: This section has been revised to include penicillin-binding protein (PBP) mutations as an additional mechanism contributing to resistance to novel β-lactam/β-lactamase inhibitor combinations.

9. Lines 297–299: This sentence does not make sense. Please revise.

Response: This sentence has been rewritten for clarity and scientific accuracy.

10. Lines 350–357: There are no capital letters in this section. Please revise.

Response: This formatting issue has been corrected, and proper capitalization has been applied throughout the section.

11. Line 418: Please briefly elaborate on the mechanism by which AMPs are able to cross the outer membrane of Gram-negative organisms.

Response: Additional text has been added to briefly explain the mechanisms by which AMPs traverse the Gram-negative outer membrane, including electrostatic interactions with lipopolysaccharides and membrane destabilization processes.

12. Line 470: amend section title to something like “bacterial resistance to AMPs” to more accurately reflect the section’s contents.

Response: The section title has been revised to more accurately reflect its focus on bacterial resistance mechanisms against AMPs.

13. Line 622: While vancomycin is technically an AMP, most clinicians and researchers would consider it an antibiotic and distinct from other AMPs such as LL37. Therefore, its inclusion in this line is confusion and should be revised or removed. Consider inserting other instances of attempted oral formulation development or briefly explaining why vancomycin is an effective representation of the barriers facing AMPs with regard to oral formulation.

Response: This section has been revised to avoid confusion regarding the classification of vancomycin. The text has been amended to clarify its inclusion in the context of oral formulation challenges, or alternatively, the reference has been removed where appropriate.

We thank the Associate Editor and the reviewers for their constructive comments. We believe that the revised manuscript adequately addresses all points raised and we look forward to further evaluation.

Sincerely,

Cesar Augusto Roque-Borda, Ph.D.

**Reviews of manuscript FEMSMC-2025-042.R1**

**Reviewer 3: Sean Van Helden, Wayne State University**

**Recommendation: Accept**

All comments were addressed adequately. No further concerns at this time.

**Reviewer 2: Daniel Propheter, The University of Texas Southwestern Medical Center**

**Recommendation: Accept**

The authors thoroughly addressed my concerns. I think the re-organization and addendums/expansions of the literature analyses tables are far more helpful to the reader. Additionally, the added commentary on the future strategies and directions on this topic are more insightful.

**Associate Editor Recommendation: Accept**

**EIC Decision: Accept**
